# Supplementary material for: µSpikeHunter: An advanced computational tool for the analysis of neuronal communication and action potential propagation in microfluidic platforms
Source: Sci Rep. 2019 Apr 8;9:5777. doi: 10.1038/s41598-019-42148-3 (PMC6453950; doi:10.1038/s41598-019-42148-3)
Supplement: Supplementary file 1 — Supplementary Figures [file 41598_2019_42148_MOESM1_ESM.pdf]

Supplementary Information

Title

µSpikeHunter: An advanced computational tool for the analysis of neuronal communication and action potential propagation in microfluidic platforms

Authors

Kristine Heiney[1,2], José C Mateus[1,2,3], Cátia DF Lopes[1,2], Estrela Neto[1,2], Meriem Lamghari[1,2], Paulo Aguiar[1,2,*]

Affiliations

[1] i3S – Instituto de Investigação e Inovação em Saúde, Universidade do Porto, Rua Alfredo Allen, 208, 4200-135 Porto, Portugal

[2] INEB – Instituto de Engenharia Biomédica, Universidade do Porto, Rua Alfredo Allen, 208, 4200-135 Porto, Portugal

[3] ICBAS - Instituto de Ciências Biomédicas Abel Salazar, Universidade do Porto, Rua Jorge de Viterbo Ferreira, 4050-313 Porto, Portugal

Corresponding Author:
Paulo de Castro Aguiar: pauloaguiar@ineb.up.pt

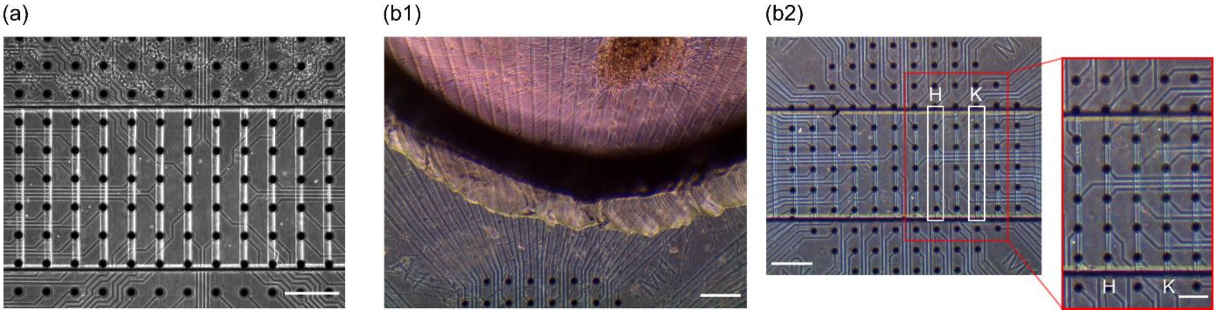

Supplementary Figure S1. Microscope images of a cortical neurons culture and a dorsal root ganglion (DRG) explant in µEF devices. (a) Cortical neurons at DIV 5 (scale bar = 200 µm). (b1) DRG explant at DIV 4 in the somal compartment. (b2) Alignment of the microchannels over the microelectrodes (scale bars = 200 µm). The inset shows a magnified view of specific microgrooves/channels, including H and K (scale bar = 100 µm).

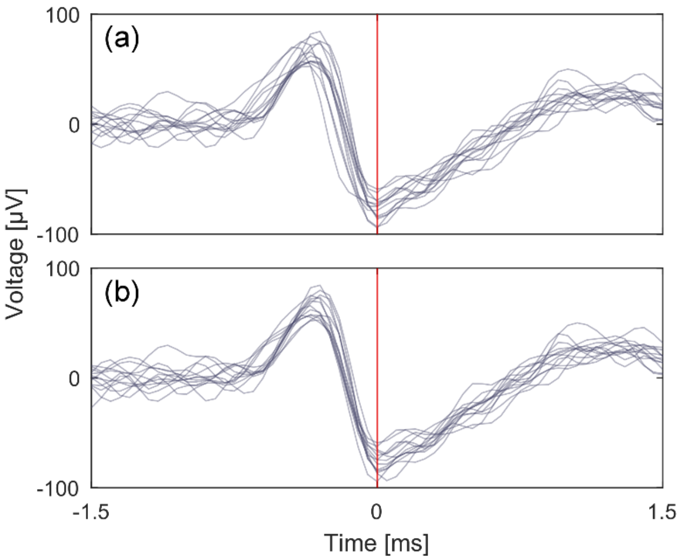

Supplementary Figure S2. Demonstration of spike realignment based on the intra-cluster cross-correlation. (a) Spikes aligned about their minima. (b) Spikes realigned based on the cross-correlation.
