# Supplementary material for: µSpikeHunter: An advanced computational tool for the analysis of neuronal communication and action potential propagation in microfluidic platforms
Source: Sci Rep. 2019 Apr 8;9:5777. doi: 10.1038/s41598-019-42148-3 (PMC6453950; doi:10.1038/s41598-019-42148-3)
Supplement: Supplementary file 2 — uSpikeHunter User Manual [file 41598_2019_42148_MOESM2_ESM.pdf]

Supplementary Information

Title

µSpikeHunter: An advanced computational tool for the analysis of neuronal communication and action potential propagation in microfluidic platforms

Authors

Kristine Heiney[1,2], José C Mateus[1,2,3], Cátia DF Lopes[1,2], Estrela Neto[1,2], Meriem Lamghari[1,2], Paulo Aguiar[1,2,*]

Affiliations

[1] i3S – Instituto de Investigação e Inovação em Saúde, Universidade do Porto, Rua Alfredo Allen, 208, 4200-135 Porto, Portugal

[2] INEB – Instituto de Engenharia Biomédica, Universidade do Porto, Rua Alfredo Allen, 208, 4200-135 Porto, Portugal

[3] ICBAS - Instituto de Ciências Biomédicas Abel Salazar, Universidade do Porto, Rua Jorge de Viterbo Ferreira, 4050-313 Porto, Portugal

Corresponding Author:
Paulo de Castro Aguiar: pauloaguiar@ineb.up.pt

µSpikeHunter: User Manual

Software for the identification and characterization of signal propagation along neuronal processes in cultures with a controlled microtopology

Kristine Heiney, Paulo Aguiar

Neuroengineering and Computational Neuroscience (NCN) Group,
i3S – Instituto de Investigação e Inovação em Saúde

Porto, Portugal

April 2018

Questions and Bug Reports:

pauloaguiar@ineb.up.pt

1 Introduction 1

2 Quick start guide to µSpikeHunter 3

2.1 Main GUI 3

2.1.1 List of objects in Main GUI 3

2.1.2 Main GUI: Step-by-step guide 6

2.2 Spike Sorting GUI 12

2.2.1 List of objects in Spike Sorting GUI 12

2.2.2 Spike Sorting GUI: Step-by-step guide 13

3 Data compatibility 16

3.1 Custom setup recording files 16

3.2 Multi Channel Systems recording files 17

4 Main GUI 18

4.1 List of objects in Main GUI 18

4.2 File and data selection panel 21

4.2.1 Data selection 21

4.2.1.1 Selection of custom setup recording files 21

4.2.1.2 Selection of Multi Channel Systems recording files 22

4.2.1.3 Selection of Multi Channel Systems data for analysis 22

4.2.2 Event detection and sending data to the analysis panel 23

4.3 Analysis panel 25

4.3.1 Event list 25

4.3.2 Voltage traces 26

4.3.3 Cross-correlation plots and single-sequence propagation velocity 26

4.3.4 Kymograph 27

4.3.5 Audio playback 28

5 Spike Sorting GUI 29

5.1 List of objects in Spike Sorting GUI 29

5.2 Spike overlays and ROI definition 31

5.3 Spike sorting process and results 33

5.4 Cluster propagation velocity estimation 35

5.5 Returning results to Main GUI 36

6 Saving files 37

6.1 Information contained in filenames 37

6.2 All analyzed data 37

6.3 All detected propagation sequences 38

6.4 Traces for a single propagation sequence 38

6.5 Spike sorting results 39

7 Glossary of terms 40

1 Introduction

Electrophysiology constitutes an important facet of neuronal communication, and recordings of neuronal electrophysiological behavior provide great insight into the manner in which they encode and transmit information. Recently, microelectrode–microfluidic (µEF) devices [1] have seen increased use in electrophysiological experiments on neurons. Such devices are composed of a microfluidic device mounted on a microelectrode array (MEA) with the microchannels of the microfluidic device aligned with the rows of microelectrodes in the MEA, as shown in Fig. 1.1. This type of device allows for the targeted observation of action potentials (APs) traveling along axons confined to the microchannels, which are too small for somata to enter. In past studies, µEF devices have been used to characterize network dynamics [2]–[4] and observe changes to the propagation velocity [5]–[7], and compartmentalized microfluidic devices without recording electrodes have also been used to observe communication between cells from different organ systems (reviewed in [8]).

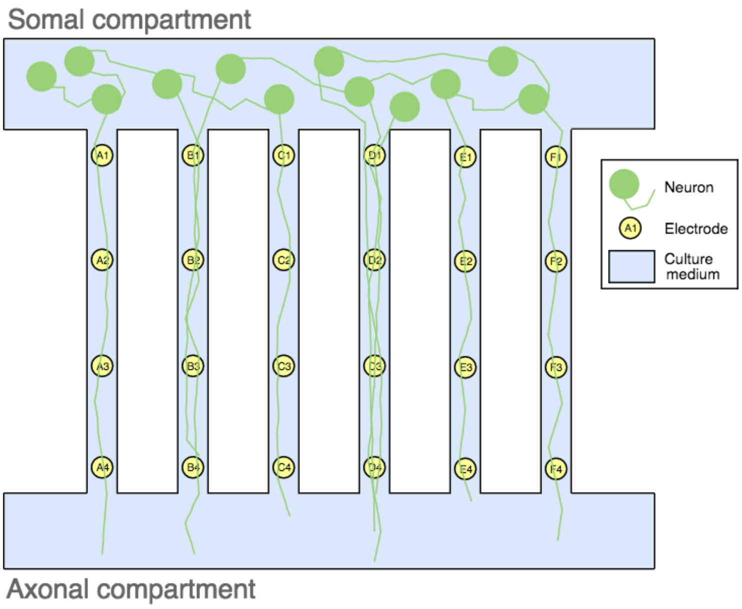

Fig. 1.1. Schematic of a µEF device with cultured neurons. The dimensions of the microchannels are such that somata are excluded but axons may grow through. The electrodes are positioned at regular intervals below the microchannels to record signals propagating along the axons confined to the microchannels.

The experimental tools necessary to obtain electrophysiological recordings using µEF devices are readily available. To complement these experimental tools, a user-friendly data analysis program called µSpikeHunter was developed for the identification and characterization of APs traveling along axons cultured in µEF devices. µSpikeHunter allows the user to readily confirm the presence of traveling waves in recorded data, determine their direction of travel, estimate their propagation velocity, and sort them into clusters based on their source.

This manual is organized as follows. A quick start guide to µSpikeHunter is first presented in Section 2, and Section 3 outlines the compatibility of µSpikeHunter with different data files. Sections 4 and 5 give a detailed explanation of how to use µSpikeHunter and describe the algorithms used in the program, and Section 6 explains how to save files with different outputs.

References

[1] J. W. Park, B. Vahidi, A. M. Taylor, S. W. Rhee, and N. L. Jeon, “Microfluidic culture platform for neuroscience research,” *Nat. Protoc.*, vol. 1, no. 4, pp. 2128–2136, 2006.

[2] L. Pan *et al.*, “An in vitro method to manipulate the direction and functional strength between neural populations,” *Front. Neural Circuits*, vol. 9, no. July, pp. 1–14, 2015.

[3] T. T. Kanagasabapathi *et al.*, “Functional connectivity and dynamics of cortical-thalamic networks co-cultured in a dual compartment device,” *J. Neural Eng.*, vol. 9, no. 3, 2012.

[4] Y. Berdichevsky, K. J. Staley, and M. L. Yarmush, “Building and manipulating neural pathways with microfluidics,” *Lab Chip*, vol. 10, no. 8, pp. 999–1004, 2010.

[5] R. Habibey, S. Latifi, H. Mousavi, M. Pesce, E. Arab-Tehrany, and A. Blau, “A multielectrode array microchannel platform reveals both transient and slow changes in axonal conduction velocity,” *Sci. Rep.*, vol. 7, no. 1, pp. 1–14, 2017.

[6] B. J. Dworak and B. C. Wheeler, “Novel MEA platform with PDMS microtunnels enables the detection of action potential propagation from isolated axons in culture,” *Lab Chip*, vol. 9, no. 3, pp. 404–410, 2009.

[7] E. Biffi *et al.*, “A microfluidic platform for controlled biochemical stimulation of twin neuronal networks,” *Biomicrofluidics*, vol. 6, no. 2, pp. 1–10, 2012.

[8] E. Neto *et al.*, “Compartmentalized Microfluidic Platforms: The Unrivaled Breakthrough of In Vitro Tools for Neurobiological Research,” vol. 36, no. 46, pp. 11573–11584, 2016.

2 Quick start guide to µSpikeHunter

This section presents a brief explanation of how to use µSpikeHunter by guiding the user through the analysis of an example file provided in conjunction with this manual. µSpikeHunter consists of two graphical user interfaces (GUIs): the Main GUI and the Spike Sorting GUI. Diagrams of the Main and Spike Sorting GUIs are presented here, and step-by-step instructions on how to use each GUI are also provided. The remainder of the user manual will refer back to the labels and names of the objects given in the diagrams.

The example file was recorded from cortical neurons at DIV 15. The sampling rate of the recording is 50 kHz, and the duration is 1 min. Screen captures of different steps of the analysis process are shown for this example file after the step-by-step guide for each GUI.

2.1 Main GUI

2.1.1 List of objects in Main GUI

This section presents a labeled diagram of the Main GUI with brief descriptions of each labeled object in the diagram. The objects in the data selection panel are labeled in red and numbered D1–D12, and those in the analysis panel are labeled in green and numbered A1–A10. Additionally, the file saving objects are labeled in purple and numbered F1–F3.

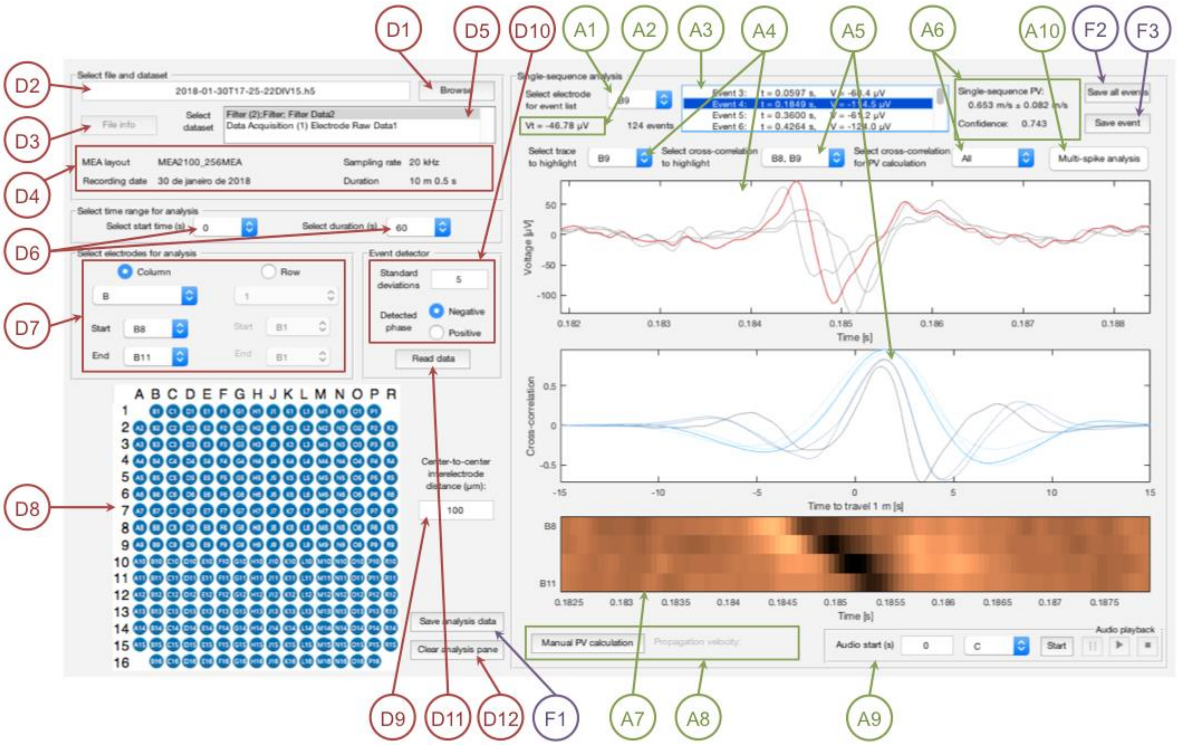

File and data selection panel

(D1) **Browse Button**: Click to select recording file (.h5, .csv, .dat, .txt)

(D2) **Filename Textbox**: Displays name of selected file

(D3) **File Information Button**: Click to display information about the file

For custom files: Displays file information in (D4)

For MCS files: Displays file information in (D4), lists recorded datasets in (D5), updates data selection lists in (D6) and (D7), and displays the electrode layout (60-, 120-, or 256-electrode layout) in (D9)

(D4) **File Information Text**: Displays MEA layout, sampling rate, and duration, along with the recording date if the file is an MCS file

(D5) **Dataset List** (MCS files only): Lists recorded datasets included in the HDF5 file

(D6) **Analysis Start Time and Duration Menus** (MCS files only): Select desired time range for analysis

(D7) **Electrode Selection Menus** (MCS files only): Select electrodes in a single row or column of the MEA for analysis

(D8) **MEA Layout Image**: Displays the MEA layout for MCS files or a MEA photograph for custom files

(D9) **Inter-Electrode Spacing Textbox**: Input the center-to-center inter-electrode distance in micrometers (default value of 100 or automatically determined for certain MCS files)

(D10) **Event Detector Parameters**: Input the number of standard deviations of the signal noise to be used as the event detector threshold and select positive or negative phase for detection

(D11) **Read Button**: Click to read the data, apply event detection, and populate the analysis pane with the results

(D12) **Clear Button**: Click to clear the analysis panel

Analysis panel

(A1) **Event Electrode Menu**: Select the electrode used to populate the Event List (A3)

(A2) **Threshold Text**: Displays the threshold used for event detection on the selected event electrode

(A3) **Event List**: Lists the events on the event electrode for each detected propagation sequence; select an event from this list for single-sequence analysis

(A4) **Voltage Trace Plot and Highlight Menu**: Plots the voltage data for all electrodes around the event selected in the event list; the electrode selected in the highlight menu is plotted in red with all other electrodes plotted in gray

(A5) **Cross-Correlation Plot and Highlight Menu**: Plots cross-correlations between all pairs of electrodes with the plots colored according to the distance between the electrode pair; the electrode pair selected in the highlight menu is plotted with full opacity with all others plotted with 35% opacity

(A6) **Single-Sequence Propagation Velocity Text and Menu**: Displays the SPV along with the confidence index for the selected event based on the electrode pair(s) selected in the drop-down menu

(A7) **Kymograph**: Displays a kymograph for the selected event

(A8) **Kymograph Propagation Velocity Tools**: Allows the user to manually estimate the propagation velocity based on a line drawn on the kymograph

(A9) **Audio Playback**: Allows the user to play an audio representation of the recorded data with 500× time dilation

(A10) **Multi-Spike Analysis Button**: Opens the Spike Sorting GUI

Saving files

(F1) **Save Analysis Data Button**: Saves all analyzed voltage and time data to a CSV file

(F2) **Save All Events Button**: Saves the times and amplitudes of the spikes on each of the electrodes for all detected propagation sequences to a CSV file

(F3) **Save Event Button**: Saves voltage and time data corresponding to the voltage trace plot in (A4) to a CSV file

2.1.2 Main GUI: Step-by-step guide

File and data selection panel: ***Custom setup files***

1. Click the Browse Button (D1) and select a data file for analysis.
2. Click the File Information Button (D3).
3. Define the inter-electrode distance in the Inter-Electrode Spacing Textbox (D9).
4. Define the number of standard deviations for the detection threshold and positive or negative phase detection in the Event Detector Parameters (D10).
5. Click the Read Button (D11).

File and data selection panel: ***MCS files***

1. Click the Browse Button (D1) and select a data file for analysis.
2. Click the File Information Button (D3).
3. Select the data stream for analysis in the Dataset List (D5).
4. Select the time range for analysis in the Analysis Start Time and Duration Menus (D6).
5. Select the desired electrode range in the Electrode Selection Menus (D7).
6. Define the inter-electrode distance in the Inter-Electrode Spacing Textbox (D9).
7. Define the number of standard deviations for the detection threshold and positive or negative phase detection in the Event Detector Parameters (D10).
8. Click the Read Button (D11).

Analysis panel

General analysis

1. Select the event electrode from the Event Electrode Menu (A1).
2. Select the event for analysis from the Event List (A3).
3. Select the voltage trace and/or cross-correlation plot to highlight from the Voltage Trace Highlight Menu (A4) and the Cross-Correlation Highlight Menu (A5).

Manual propagation velocity estimation

1. Click the “Manual PV calculation” button in the Kymograph Propagation Velocity Tools (A8).
2. Draw a line as instructed in Section 4.3.4 and adjust the ends as needed.
3. Once the line is finalized, double click to see the manual propagation velocity estimate.

Audio playback

1. Define the desired start time in the textbox in the Audio Playback (A9).
2. Select the desired chord from the drop-down menu.
3. Click the “Start” button.
4. The playback may be paused, resumed, and stopped by clicking the corresponding buttons in the Audio Playback (A9).

Open Spike Sorting GUI

1. Click the Multi-Spike Analysis Button (A10).
2. See Section 2.2.2 for a step-by-step guide on how to use the Spike Sorting GUI.

Save files

1. Click the Save All Events Button (F2) to save a CSV file with all the voltage data for each of the selected electrodes along with the corresponding time stamps.
2. Click the Save Event Button (F3) to save a CSV file with the voltage data for the event currently selected in the Event List (A3) along with the corresponding time stamps.

File and data selection panel: *MCS files*

Opening screen

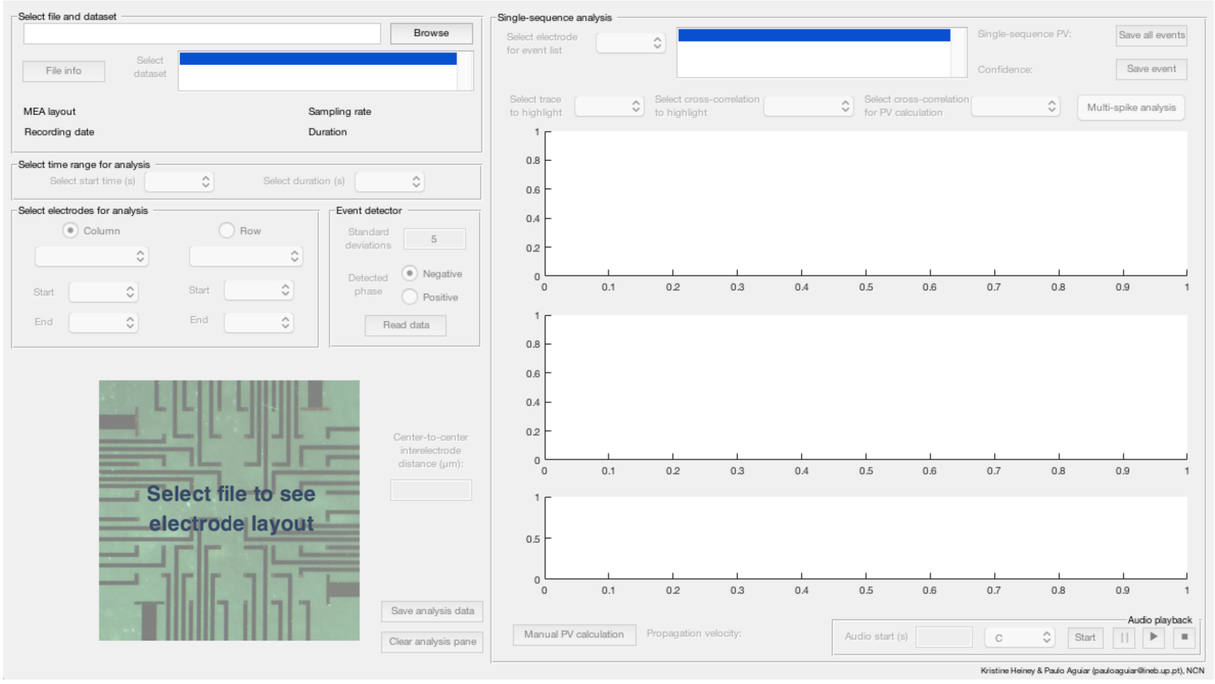

Select the example file for analysis

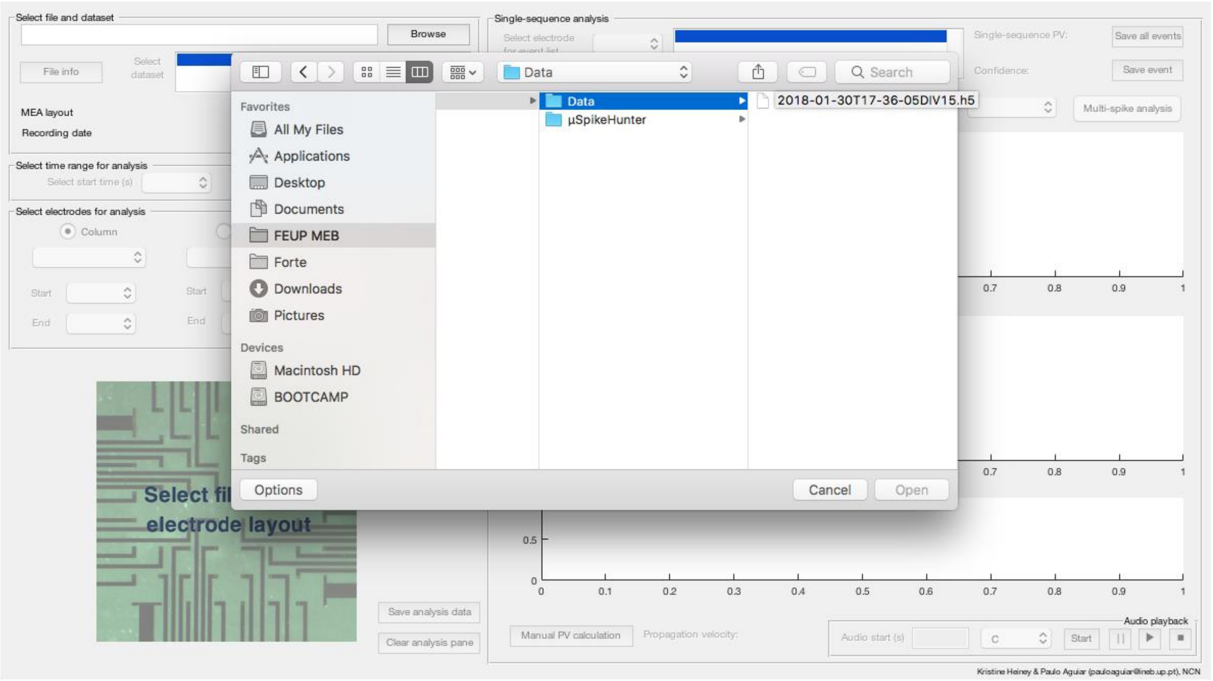

Click the File Information Button (D3)

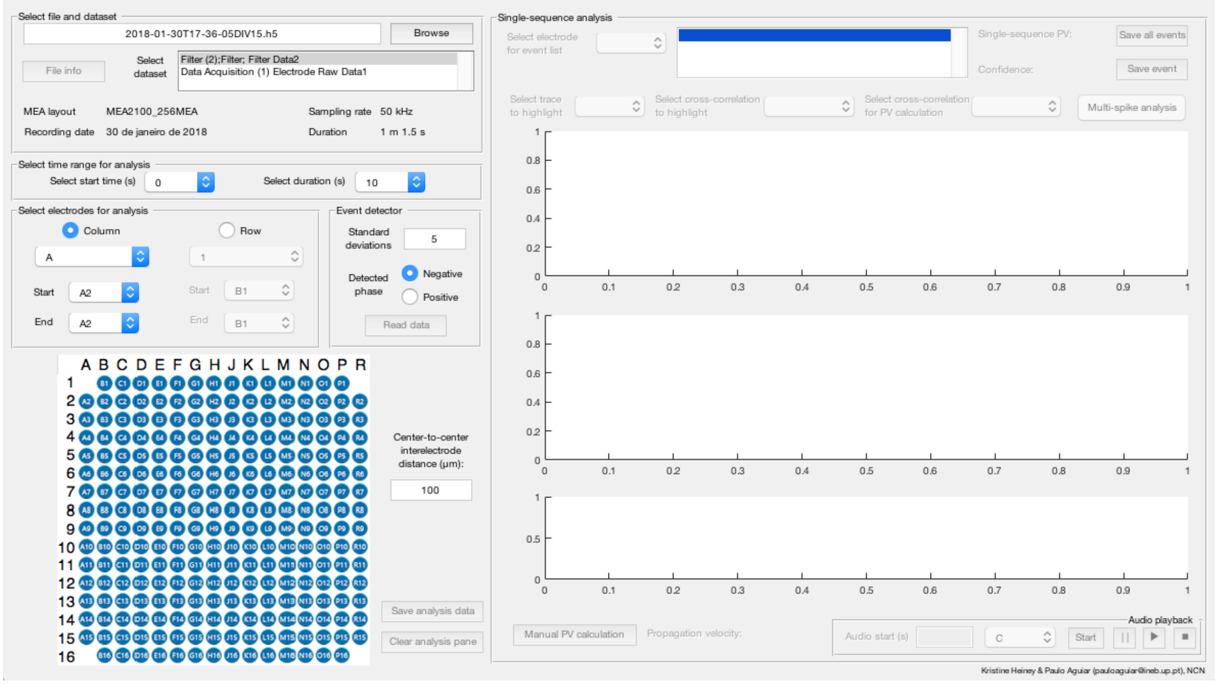

Select the data parameters as shown below

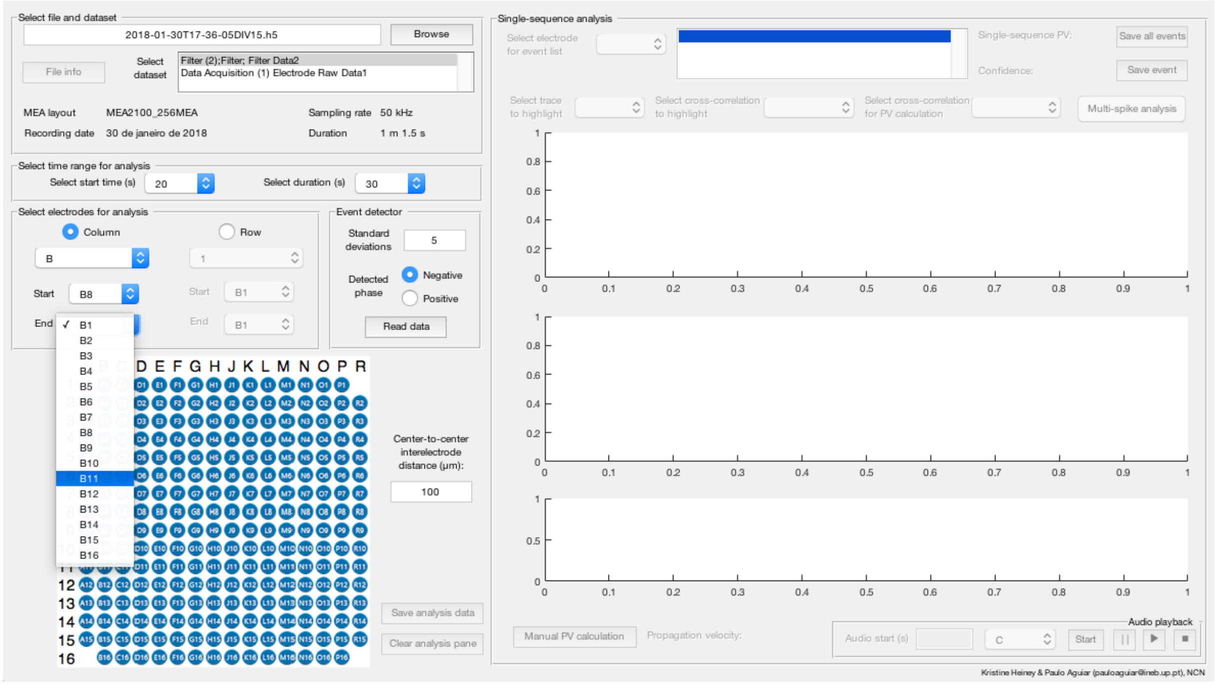

Click the Read Button (D11)

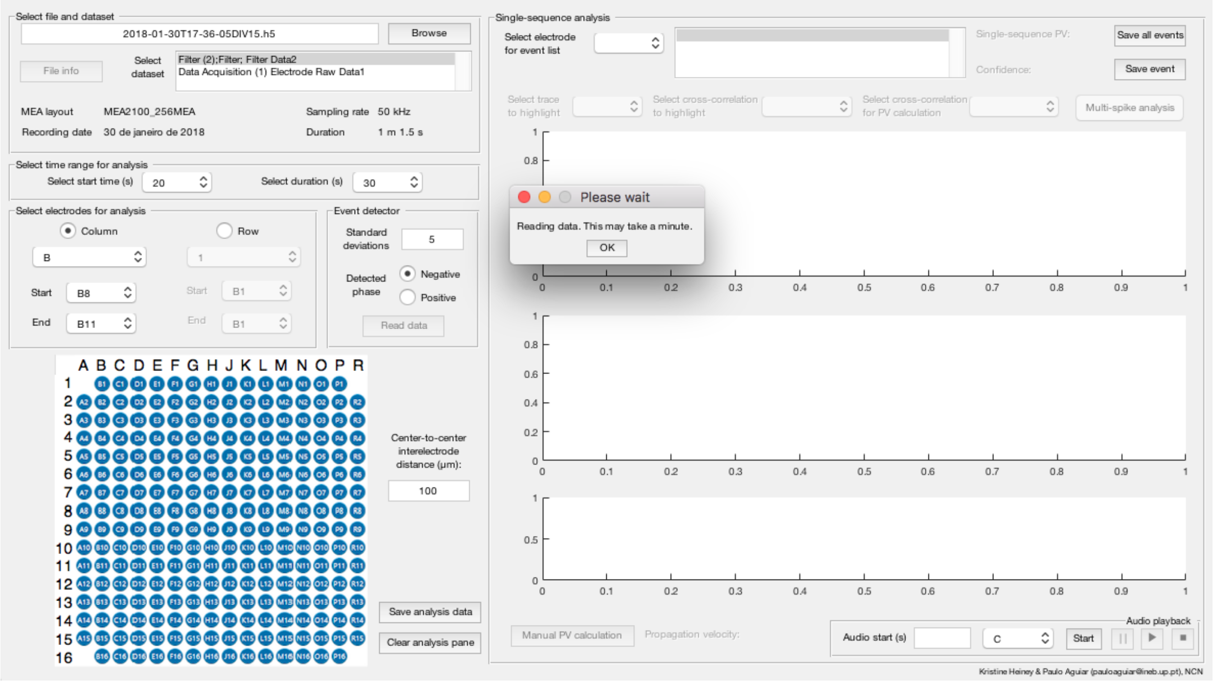

Analysis panel

General analysis

The plots and SPV estimates can be viewed for any event selected from the Event List (A3)

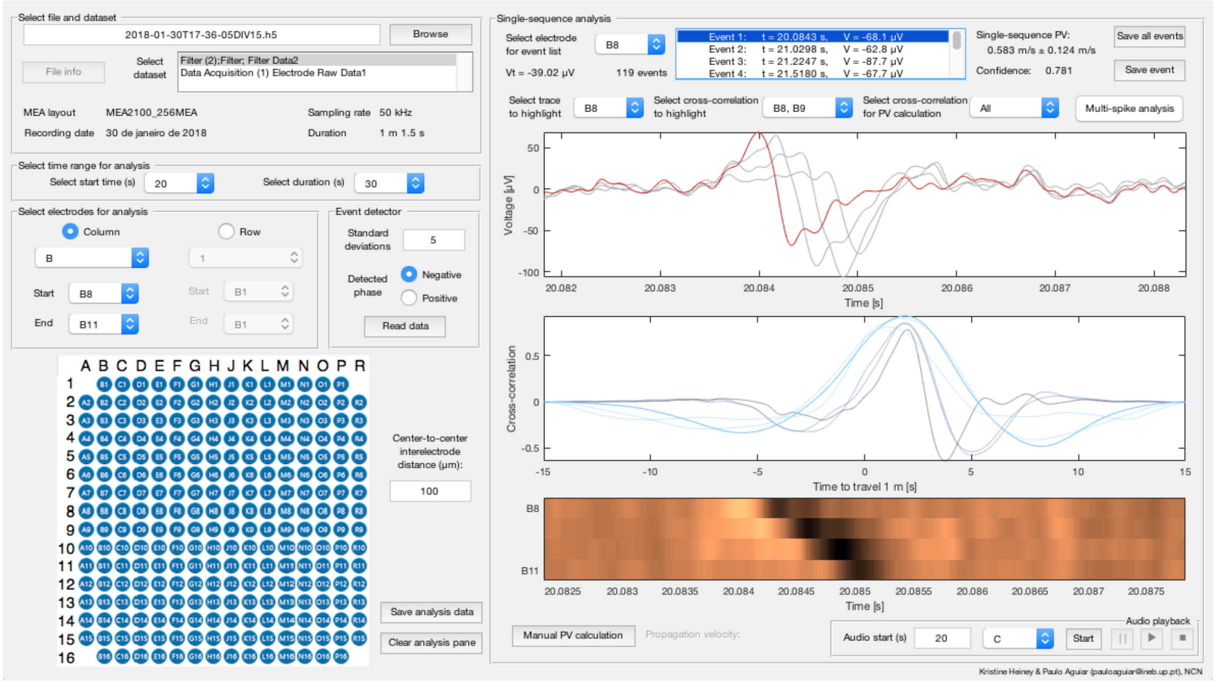

Manual propagation velocity estimation

The line for the manual propagation velocity estimate should be drawn as shown below

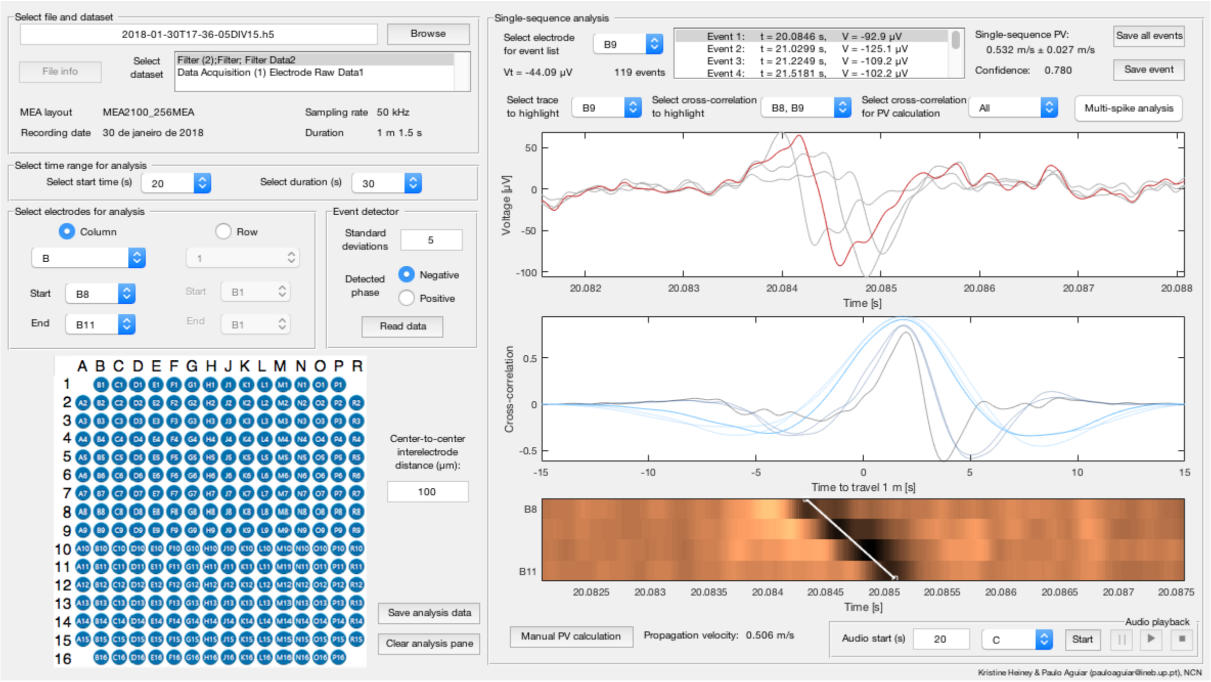

Audio playback

Set the start time and select the desired chord in the Audio Playback (A9)

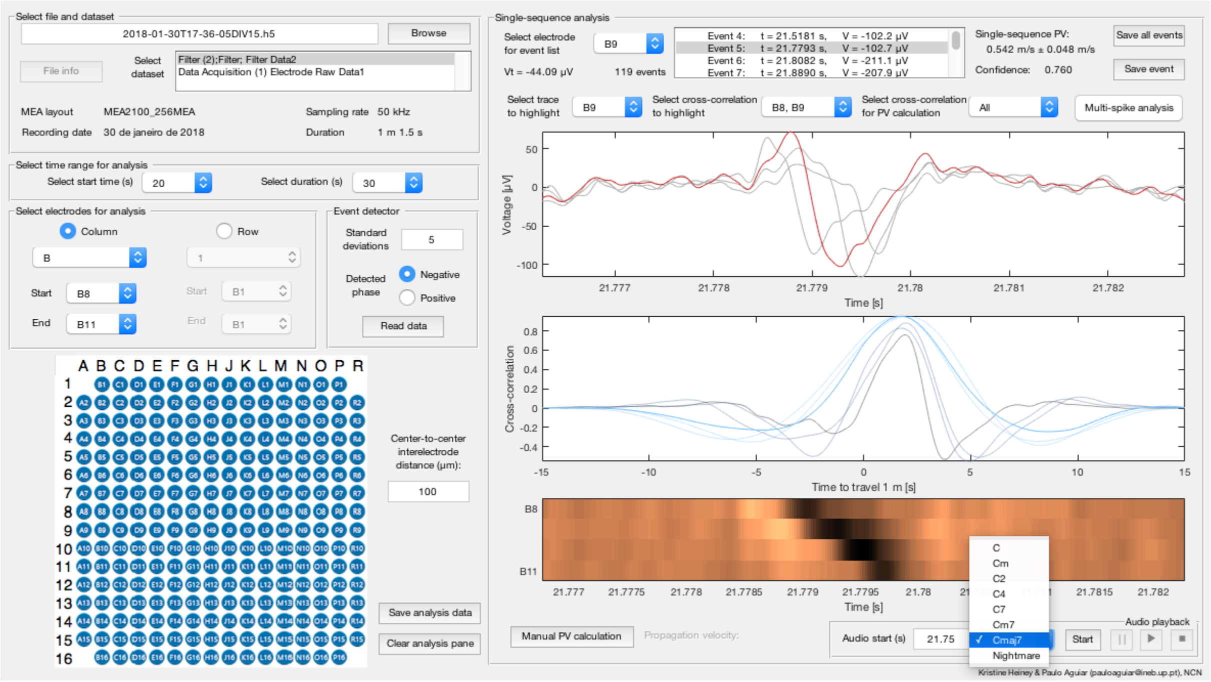

Click the "Start" button to start the audio playback and pause, resume, and stop the playback as desired

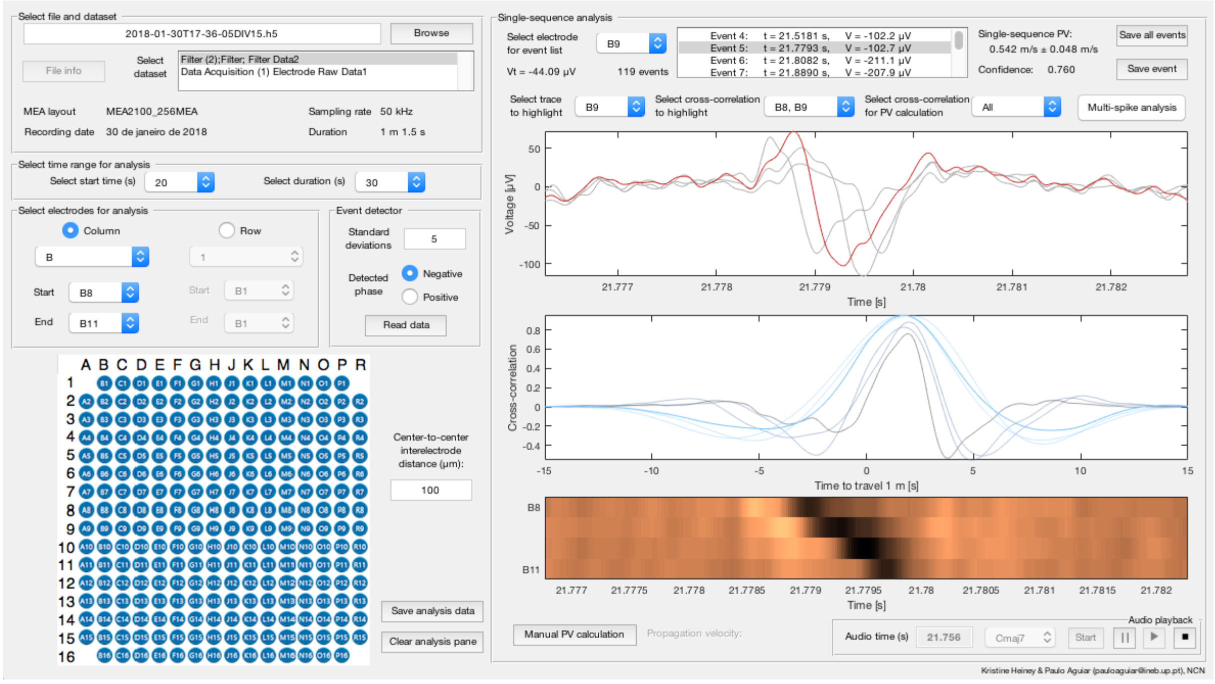

2.2 Spike Sorting GUI

2.2.1 List of objects in Spike Sorting GUI

This section presents a labeled diagram of the Spike Sorting GUI with brief descriptions of each labeled object in the diagram. The objects in the Spike Sorting are labeled in orange and numbered S1–S7, and the file saving object is labeled in purple and numbered F4.

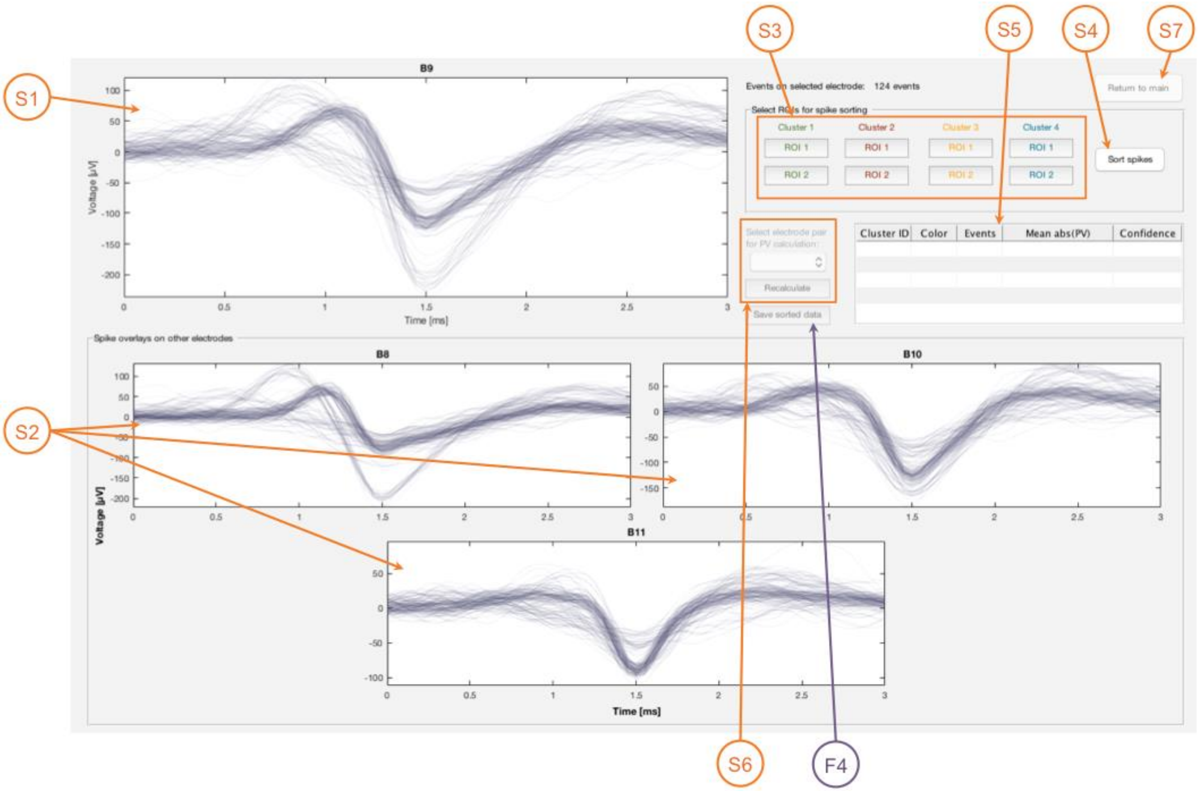

Spike Sorting GUI

(S1) **Event Electrode Spike Overlay Plot**: Shows a plot of all the events on the event electrode detected as part of propagation sequences aligned based on their minima

(S2) **Other Electrode Spike Overlay Plots**: Show plots of events corresponding to the events detected on the event electrode on each of the other electrodes

(S3) **ROI Definition Tools**: Allow the user to draw one or two ROIs for up to four clusters to sort the plotted events

(S4) **Sort Spikes Button**: Sorts the events into source clusters based on the defined ROIs and outputs the results into the Sorting Results Table

(S5) **Sorting Results Table**: Displays the results of the spike sorting, including the cluster ID, plotting color, number of events, and mean propagation speed for each cluster

(S6) **Cluster Propagation Velocity Electrode Selection**: Allows the user to select the electrode pair for calculating the CPV

(S7) **Return to Main GUI Button**: Sends cluster IDs and CPV estimates back to the Event List (A3) in the Main GUI

Saving files

(F4) **Save Sorted Data Button**: Saves a CSV file per source cluster with the spike times on each electrode, the CPV, the SPV, and the CPV and SPV confidence indices

2.2.2 Spike Sorting GUI: Step-by-step guide

Define ROIs

1. Click on any of the ROI buttons in the ROI Definition Tools (S3).
2. Draw a rectangular ROI by clicking and dragging on the Event Electrode Spike Overlay Plot (S1).
3. Adjust the bounds of the ROI by clicking and dragging on any of the edges or corners of the ROI.
4. Repeat for as many clusters as desired with up to two ROIs defining the conditions for each of the clusters. The ROIs can be defined in any order and for any number of clusters.
5. To delete an ROI, right click on the ROI and select “Delete” from the menu.
6. If an ROI has already been drawn and the corresponding button for that ROI is clicked again, the ROI is deleted and a new version of it can be drawn.

Sort spikes and display results

1. Click the Sort Spikes Button (S4).
2. To use electrodes other than the most distant pair for CPV calculation, select the desired electrode pair from the Cluster Propagation Velocity Electrode Selection (S6) and click the “Recalculate” button.

Return spike sorting results to Main GUI

1. Click the Return to Main GUI Button (S7).
2. View the sorting and CPV estimation results in the Event List (A3) in the Main GUI.

Save sorting results

1. Click the Save Sorted Data Button (F4) to save a CSV file for each source cluster with information about each propagation sequence in that cluster: the spike times on each electrode, the CPV estimate for the sequence, the SPV estimate for the sequence, and the confidence indices for the two propagation velocity estimates.

Spike Sorting GUI

In the example presented below, B9 was selected as the event electrode in the Event Electrode Selection Menu (A1) in the Main GUI. Open the Spike Sorting GUI from the Main GUI by clicking the Multi-Spike Analysis Button (A10).

Define ROIs

Draw ROIs on the Event Electrode Spike Overlay Plot (S1) as shown below using the ROI Definition Tools (S3)

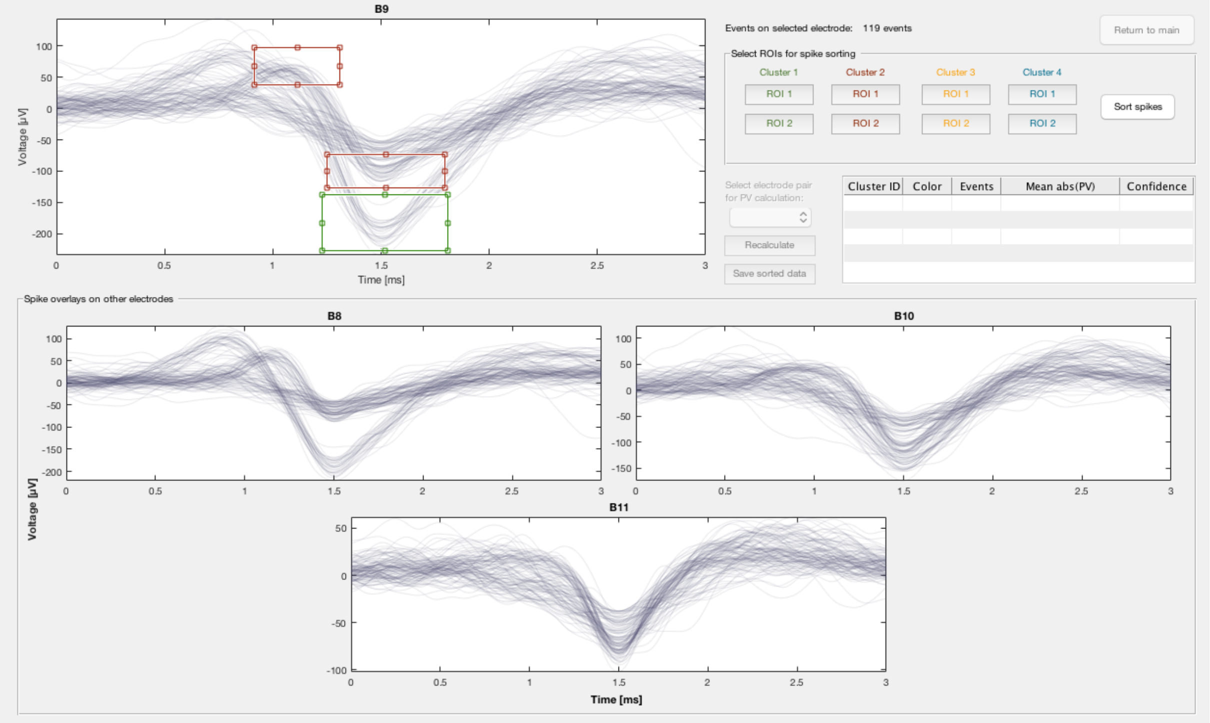

Sort spikes and display results

Click the Sort Spikes Button (S4)

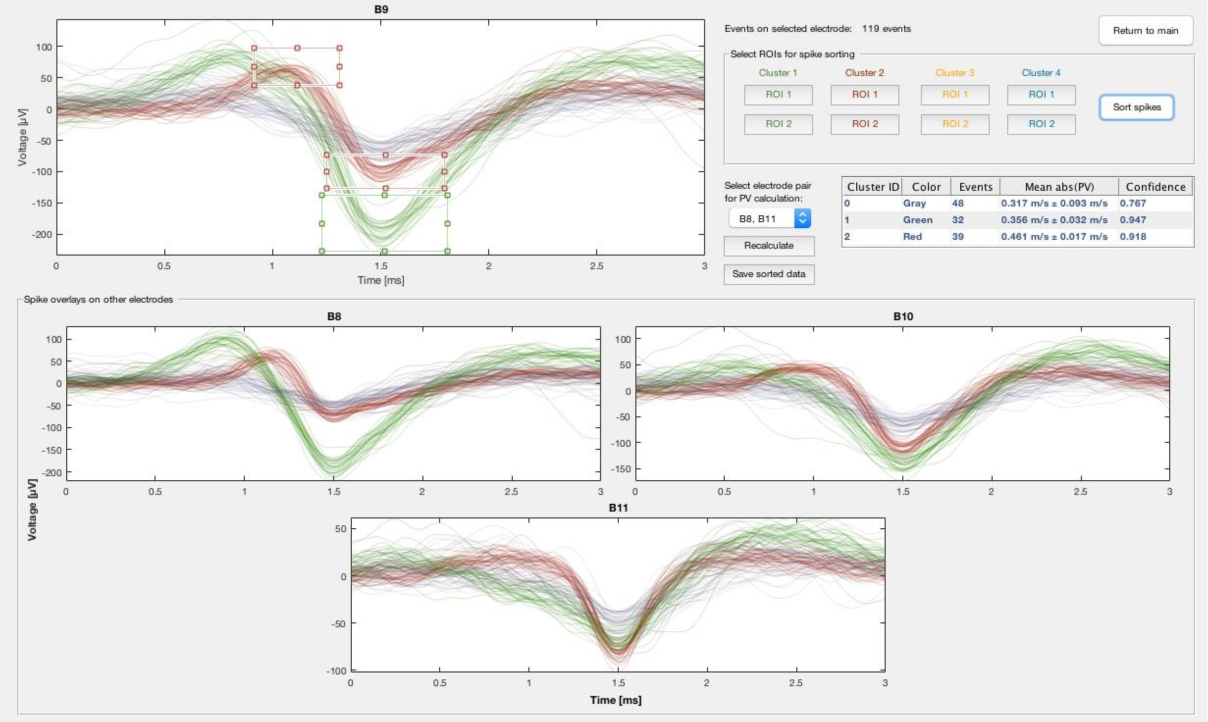

Return spike sorting results to Main GUI

Click the Return to Main GUI Button (S7) to see the Event List (A3) updated with the cluster ID and CPV estimate for each spike

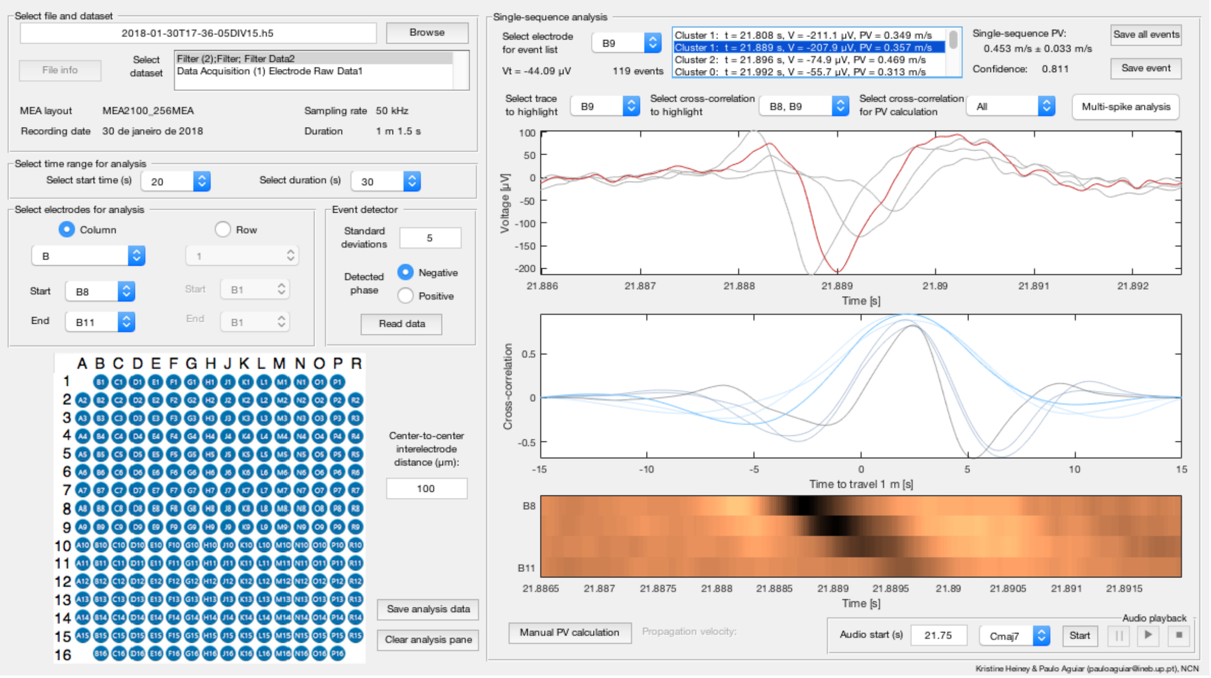

3 Data compatibility

µSpikeHunter is compatible with recordings obtained using both custom µEF setups and the commercial MCS MEA2100 systems (Multi Channel Systems GmbH, Reutlingen, Germany). This section provides guidance on how to format custom files to be read by µSpikeHunter and which MCS recording systems can be used in conjunction with µSpikeHunter.

It should be noted that µSpikeHunter does not contain any data filtering tools. Thus, it is recommended that the user filter the data prior to importing it for analysis in µSpikeHunter. The example data shown in this manual have been filtered with a low-pass Butterworth filter with a cutoff frequency of 4 kHz followed by a high-pass Butterworth filter with a cutoff frequency of 300 Hz. Filtering was performed using the MCS recording software Multi Channel Experimenter. Additionally, it is recommended that a sampling frequency of at least 10 kHz be used when taking recordings.

3.1 Custom setup recording files

The user may load data in CSV format from recordings obtained using a custom recording setup. This data should be formatted in columns with the first column corresponding to the time in milliseconds and all subsequent columns corresponding to the recorded voltages in microvolts for each electrode to be analyzed, progressing from one electrode to the next inside a single microchannel of the µEF device. An example of the organization that should be used is shown in Fig. 3.1. This example shows 1 ms of recorded data on four electrodes. µSpikeHunter can handle data from up to 16 electrodes with a uniform inter-electrode spacing.

	Time (ms)	A1 (µV)	A2 (µV)	A3 (µV)	A4 (µV)
	1	2	3	4	5
1	0	4.7700	4.3200	5.6800	8.6400
2	0.0500	14.7700	16.2500	19.8900	27.7300
3	0.1000	18.0700	26.0200	30.2300	36.9300
4	0.1500	11.7000	27.3900	27.9500	31.2500
5	0.2000	5.6800	23.0700	16.9300	23.1800
6	0.2500	6.0200	14.6600	7.3900	17.6100
7	0.3000	9.0900	5.9100	5	14.2000
8	0.3500	10.4500	3.8600	5.4500	12.2700
9	0.4000	10.4500	6.4800	3.3000	10.5700
10	0.4500	10.8000	6.3600	0.2300	8.4100
11	0.5000	10.5700	4.6600	2.0500	5.1100
12	0.5500	7.5000	6.1400	7.6100	1.1400
13	0.6000	2.3900	10.2300	9.5500	-1.3600
14	0.6500	-0.4500	12.6100	6.7000	-2.3900
15	0.7000	-0.6800	10.4500	4.0900	-5.9100
16	0.7500	-2.5000	7.1600	2.5000	-12.5000
17	0.8000	-3.9800	4.7700	0.6800	-17.5000
18	0.8500	-3.7500	-0.4500	-1.3600	-15.9100
19	0.9000	-2.7300	-7.9500	-2.9500	-9.6600
20	0.9500	-4.3200	-10.4500	-3.6400	-4.5500
21	1	-10.2300	-6.5900	-5.1100	-3.4100

Fig. 3.1. Data recorded on four electrodes formatted for analysis in µSpikeHunter. The time values are given in milliseconds, and the voltages are given in microvolts. The columns are labeled to correspond to the electrodes in one of the microchannels in the example µEF configuration shown in Fig. 1.1.

It is suggested that the second column correspond to the electrode at the most proximal end of the microchannel and the final column to that at the most distal end; this will yield results in the GUI with forward propagation corresponding to propagation from the soma to the axon terminal. If the example data in Fig. 3.1 correspond to the third microchannel from the left in the example setup shown in Fig. 1.1, this would mean that columns 2–5 in the CSV file represent data from electrodes C1–C4, respectively.

3.2 Multi Channel Systems recording files

µSpikeHunter is compatible with HDF5 files generated from MCS recordings obtained using the MCS MEA2100-60-System, MEA2100-120-System, and MEA2100-256-System, which can be used in conjunction with 60-, 120-, and 256-electrode MEAs produced by MCS. Once recordings have been obtained using the MCS recording software Multi Channel Experimenter, they can be converted to HDF5 files using the MCS data handling software Multi Channel DataManager. Users are directed to the MCS documentation on the corresponding equipment and software for instructions on data recording and conversion.

4 Main GUI

This section presents the capabilities of the Main GUI of µSpikeHunter. This GUI allows the user to select the data for analysis and analyze the data at the single-propagation-sequence level. The Main GUI consists of two panels: the file and data selection panel and the analysis panel. The functionality of each item in the two panels, except for the buttons to save files, is described in this section.

4.1 List of objects in Main GUI

This section shows again the labeled diagram of the Main GUI with brief descriptions of each labeled object in the diagram. The objects in the data selection panel are labeled in red and numbered D1–D12, and those in the analysis panel are labeled in green and numbered A1–A10. Additionally, the file saving objects are labeled in purple and numbered F1–F3.

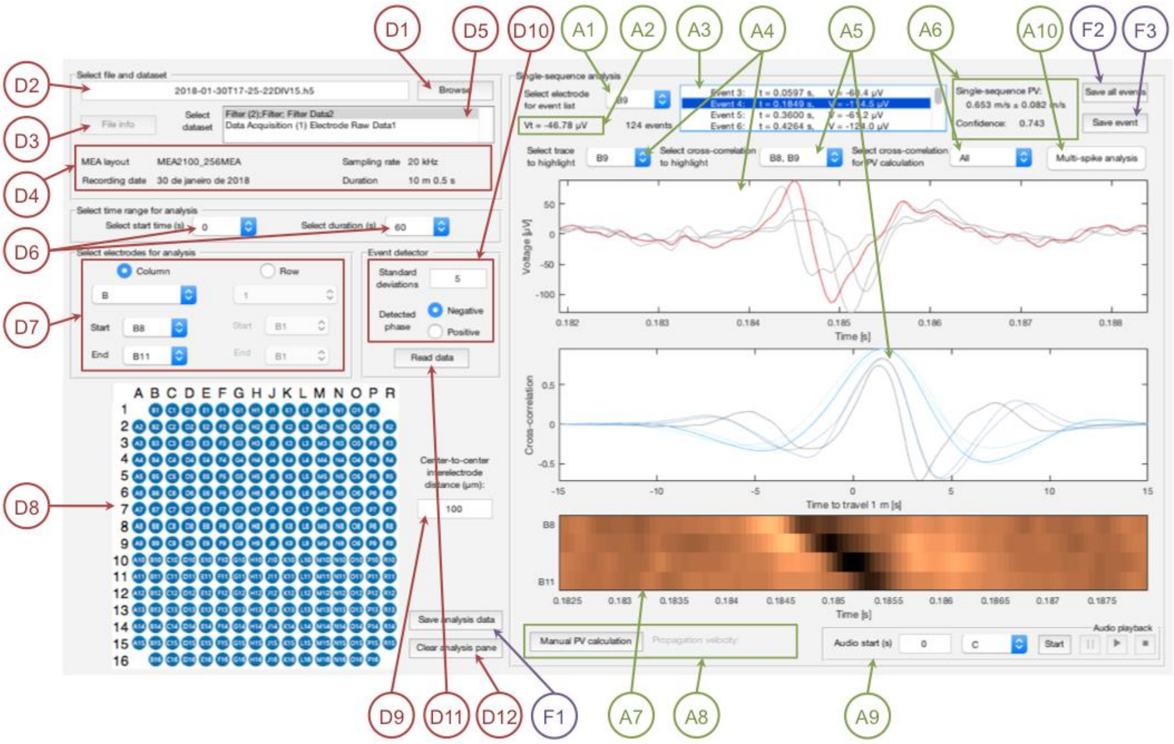

File and data selection panel

(D1) **Browse Button**: Click to select recording file (.h5, .csv, .dat, .txt)

(D2) **Filename Textbox**: Displays name of selected file

(D3) **File Information Button**: Click to display information about the file

For custom files: Displays file information in (D4)

For MCS files: Displays file information in (D4), lists recorded datasets in (D5), updates data selection lists in (D6) and (D7), and displays the electrode layout (60-, 120-, or 256-electrode layout) in (D9)

(D4) **File Information Text**: Displays MEA layout, sampling rate, and duration, along with the recording date if the file is an MCS file

(D5) **Dataset List** (MCS files only): Lists recorded datasets included in the HDF5 file

(D6) **Analysis Start Time and Duration Menus** (MCS files only): Select desired time range for analysis

(D7) **Electrode Selection Menus** (MCS files only): Select electrodes in a single row or column of the MEA for analysis

(D8) **MEA Layout Image**: Displays the MEA layout for MCS files or a MEA photograph for custom files

(D9) **Inter-Electrode Spacing Textbox**: Input the center-to-center inter-electrode distance in micrometers (default value of 100 or automatically determined for certain MCS files)

(D10) **Event Detector Parameters**: Input the number of standard deviations of the signal noise to be used as the event detector threshold and select positive or negative phase for detection

(D11) **Read Button**: Click to read the data, apply event detection, and populate the analysis pane with the results

(D12) **Clear Button**: Click to clear the analysis panel

Analysis panel

(A1) **Event Electrode Menu**: Select the electrode used to populate the Event List (A3)

(A2) **Threshold Text**: Displays the threshold used for event detection on the selected event electrode

(A3) **Event List**: Lists the events on the event electrode for each detected propagation sequence; select an event from this list for single-sequence analysis

(A4) **Voltage Trace Plot and Highlight Menu**: Plots the voltage data for all electrodes around the event selected in the event list; the electrode selected in the highlight menu is plotted in red with all other electrodes plotted in gray

(A5) **Cross-Correlation Plot and Highlight Menu**: Plots cross-correlations between all pairs of electrodes with the plots colored according to the distance between the electrode pair; the electrode pair selected in the highlight menu is plotted with full opacity with all others plotted with 35% opacity

(A6) **Single-Sequence Propagation Velocity Text and Menu**: Displays the SPV along with the confidence index for the selected event based on the electrode pair(s) selected in the drop-down menu

(A7) **Kymograph**: Displays a kymograph for the selected event

(A8) **Kymograph Propagation Velocity Tools**: Allows the user to manually estimate the propagation velocity based on a line drawn on the kymograph

(A9) **Audio Playback**: Allows the user to play an audio representation of the recorded data with 500× time dilation

(A10) **Multi-Spike Analysis Button**: Opens the Spike Sorting GUI

Saving files

(F1) **Save Analysis Data Button**: Saves all analyzed voltage and time data to a CSV file

(F2) **Save All Events Button**: Saves the times and amplitudes of the spikes on each of the electrodes for all detected propagation sequences to a CSV file

(F3) **Save Event Button**: Saves voltage and time data corresponding to the voltage trace plot in (A4) to a CSV file

4.2 File and data selection panel

The file and data selection panel allows the user to select the file containing the recording data; set the event detection parameters; define the inter-electrode distance; and, in the case of MCS files, select the desired electrodes and timespan for analysis. Once all these parameters have been set, the user may then send the data to the analysis pane. This section describes this procedure for both custom setup recording files and MCS recording files.

***Note**: Changing any parameters in the data selection panel will automatically clear the analysis panel.*

4.2.1 Data selection

This section describes the process of selecting the data to be analyzed in the case where custom and MCS setups are used for recording.

4.2.1.1 Selection of custom setup recording files

If a custom recording setup is used, the user may import a CSV file containing the data for up to 16 electrodes contained within a single microchannel of the µEF device. µSpikeHunter supports files with file extensions of .csv, .dat, and .txt. The data should be formatted as described in Section 3.1. As stated in Section 3, µSpikeHunter does not contain any filtering tools, and imported data should thus be filtered prior to analysis in this program.

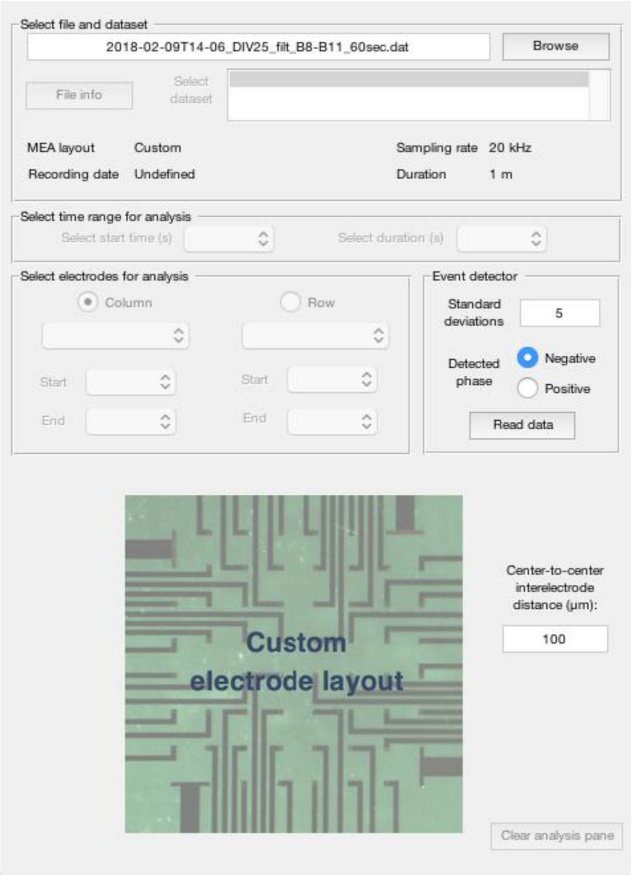

Fig. 4.1. Appearance of the file and data selection panel when a custom setup recording file is selected for analysis.

The desired file can be selected by clicking the Browse Button (D1) and navigating to the file in the file selection window. Once the data file has been selected, the filename will appear in the Filename Textbox (D2). Clicking the File Info Button (D3) will then list the sampling rate and the recording duration for the data contained in the file in the File Information Text (D4).

Before analysis, the user must input the inter-electrode distance into the Inter-Electrode Spacing Textbox (D9). Figure 4.1 shows an example of the appearance of the file and data selection panel when a custom setup recording file has been selected for analysis.

4.2.1.2 Selection of Multi Channel Systems recording files

µSpikeHunter is compatible with data files recorded using Multi Channel Experimenter in conjunction with MCS MEA2100-Systems with 60-, 120-, or 256-electrode MEAs and converted to HDF5 files using Multi Channel DataManager, as described in Section 3.2. Users are directed to the MCS documentation on the corresponding equipment and software for instructions on data recording and conversion. As stated in Section 3, µSpikeHunter does not contain any filtering tools, and imported data should thus be filtered prior to analysis in this program. Multi Channel Experimenter and Multi Channel Analyzer both provide the ability to filter raw data recorded using the MCS MEA2100-Systems.

Once the recording file has been selected, clicking the File Info Button (D3) will then list the sampling rate and the recording duration for the data contained in the file as well as the MEA system used for the recording and the recording date in the File Information Text (D4). A list of data streams (e.g., raw data, filtered data) is also provided in the Dataset List (D5). It is recommended that the user select the filtered data stream for analysis.

4.2.1.3 Selection of Multi Channel Systems data for analysis

Clicking the File Info Button (D3) also populates the data selection menus, which enable a limited set of recorded data to be selected for analysis. Data recorded using MEAs with a large number of electrodes can be cumbersome to handle, and this selection capability allows the user to control the amount of data for analysis to prevent overly long computation times. The start time and duration and duration of the data for analysis can be defined in the Analysis Start Time and Duration Menus (D6). The start times can be selected in increments of 10 s, and the analysis duration may range from 10 s to 10 min.

Beneath the Analysis Start Time and Duration Menus (D6), the user may select the range of electrodes for analysis in the Electrode Selection Menus (D7). The electrode layout for the selected file is shown in the MEA Layout Image (D8) to provide the user with visual feedback regarding the electrode positioning. The inter-electrode spacing in micrometers must then be provided in the Inter-Electrode Spacing Textbox (D9). The default value for the inter-electrode spacing is 100 µm. For some MCS recordings, this value is able to be automatically detected; however, the user should always check to ensure this value is correct.

Because µSpikeHunter is intended for use with µEF devices or other similar recording setups with structured topologies, the electrodes to be selected for analysis in the Electrode Selection Menus (D7) must be within a single row or column of electrodes in the MEA. These

selected electrodes should correspond to the electrodes positioned beneath a single microchannel of the µEF device.

In the example shown in Fig. 1.1, the user would select the "Column" radio button and could then select any of the column names for analysis; for example, column A could be selected to analyze the electrodes in the leftmost microchannel. It is suggested that the start electrode be at the most entrance of the microchannel most proximal to the somal compartment and the end electrode the most distal. In this case, A1 would then be the start electrode and A4 the end electrode. This selection choice yields results in the GUI with forward propagation corresponding to propagation from the soma to the axon terminal.

4.2.2 Event detection and sending data to the analysis panel

Before sending the data to the analysis panel, the Event Detector Parameters (D11) must be set. The number of standard deviations used to define the detection threshold is input into the textbox, and the detected phase is set using the radio buttons.

The event detector uses a two-step process to determine the threshold for event detection. First, outliers are eliminated by excluding any data points that are more than three scaled median absolute deviations from the median of the recorded signal. The remaining data points in the selected time range (Analysis Start Time and Duration Menus (D6)) are considered to represent the signal noise. With the outliers excluded, the median and standard deviation of the signal are then obtained. The detection threshold is defined as the given number of standard deviations above or below the median for this second calculation. During analysis, the detection threshold for the event electrode can be found in the Detection Threshold Text (A2).

Clicking the Read Button (D11) applies the event detector to one of the electrodes selected for analysis in the Electrode Selection Menus (D7) and obtains the times for each of the events on that electrode. The detection thresholds are also calculated for each of the other electrodes. A detected event is considered to be part of a propagation sequence if three criteria are met, as described below.

First, event detection is applied to the other electrodes, but only within a specific temporal search window. The search window on each electrode is defined for each of the detected events based on the distance to the electrode on which event detection is applied. This search window is defined to correspond to an AP traveling at a minimum propagation velocity of ±0.1 m/s. For example, for three electrodes with an inter-electrode distance of 100 µm, the maximum time delay between spikes recorded on the first and second or second and third electrodes would be 1 ms, and that between spikes on the first and third electrodes would be 2 ms. The first criterion is met for a given event if there are events on every other electrode within the as-defined search window.

Second, to deal with cases in which instrument noise produces simultaneous or near-simultaneous peaks on all electrodes, the times of the events on the most distant electrodes in the range of electrodes selected for analysis are compared. The second criterion is met

when the lag between these two event times corresponds to a propagation velocity that is below 100 m/s.

Third, to ensure the propagation direction is consistent throughout the propagation sequence, the Kendall rank coefficient of the electrode number and the spike arrival time on each electrode is obtained. The third criterion is met when the Kendall coefficient is at least 0.8.

Once all of the propagation sequences have been obtained, the results are sent to the data analysis panel for visualization and analysis. The analysis panel can be cleared by clicking the Clear Button (D12).

4.3 Analysis panel

The analysis panel of the Main GUI allows the user to select an event from the list of detected events on the selected event electrode and view and interact with a number of analysis tools for the propagation sequence corresponding to that event, including voltage traces, inter-electrode cross-correlations, a kymograph, and an audio playback tool. This section describes the information presented in each of the analysis objects in the analysis panel and how to use each object. This section refers back to the labels given in the diagram on page 18, and a separate image of the analysis panel is shown in Fig. 4.2 to show a detailed view of this panel.

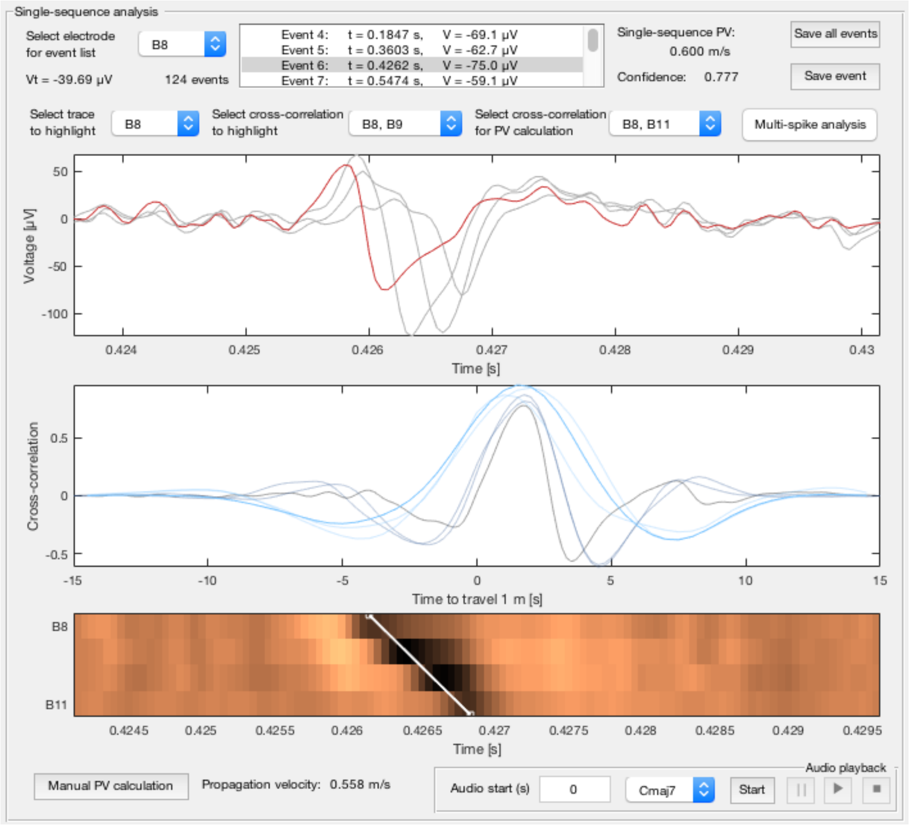

Fig. 4.2. Analysis panel of Main GUI. See the diagram on page 18 for names and labels of analysis objects.

This analysis panel allows data analysis on the single-sequence level. To perform multi-sequence analysis, the user may click the Multi-Spike Analysis Button (A10), which opens the Spike Sorting GUI in a separate window. For a description of the capabilities of the Spike Sorting GUI and instructions on how to use it, see Section 5.

4.3.1 Event list

The Event List (A3) lists all events on the event electrode that are part of detected propagation sequences. The event electrode can be set by selecting the desired electrode from the Event Electrode Menu (A1). The detection threshold for the event electrode is displayed in the Threshold Text (A2), and the total number of propagation sequences is also listed below the Event Electrode Menu (A1). Each event in the Event List (A3) gives the event number, the time at which the peak voltage occurs, and the peak voltage value.

Selecting an event from this list allows the user to perform single-sequence analysis on the propagation sequence corresponding to the selected event.

4.3.2 Voltage traces

The Voltage Trace Plot (A4) shows a plot of the voltage traces on all electrodes within a time window defined based on the time of the selected event in the Event List (A3). All plots are shown in gray except for the trace selected in the Voltage Trace Highlight Menu (A4), which is highlighted in red. From this plot, the user is often able to visually determine whether the propagation sequence shows anterograde or retrograde propagation.

4.3.3 Cross-correlation plots and single-sequence propagation velocity

The Cross-Correlation Plot (A5) shows plots of inter-electrode cross-correlations for all possible electrode pairs for the selected electrodes. The plots are colored according to the distance between the electrode pair, with the lightest color corresponding to the closest pairs (neighboring electrodes) and the darkest to the most distant pair (the start and end electrodes defined in the Electrode Selection Menus (D7)). The electrode pair selected in the Cross-Correlation Highlight Menu (A5) is plotted with full opacity, and all other cross-correlations are plotted with 35% opacity.

The cross-correlation values are normalized so that all autocorrelations take a unit value at zero lag. The lag timescale of the cross-correlation is also normalized with respect to the distance between the electrodes for which the cross-correlation is being performed; the normalized lags thus correspond to the time in seconds it would take an event to travel 1 m. This means that the horizontal axis of the cross-correlation is equivalent to the inverse of the propagation velocity of a spike for each plotted time lag; thus, under the assumption that the propagation velocity is constant across all electrodes, the peaks of all cross-correlations should be aligned.

The single-sequence propagation velocity (SPV) is displayed in the Single-sequence Propagation Velocity Text (A6) along with the confidence index for the estimate. By default, the SPV is calculated as the average propagation velocity obtained for each electrode pair, which is calculated by determining the time at which each cross-correlation achieves a maximum value and calculating the speed from this time and the distance between the two electrodes. The SPV is presented along with the standard deviation of the velocities obtained with the different electrode pairs. The confidence index is the peak value of the autocorrelation-normalized cross-correlation with the lowest peak among all of the electrode pairs. In the case of the propagation sequence shown in Fig. 4.2, this value is the peak value of the black curve, which is the cross-correlation between the two most distant electrodes (B8 and B11). However, when one of the cross-correlations takes a peak at time zero, this causes the average over all the electrode pairs to be indeterminate. Therefore, the user can select the desired electrode pair from the Single-sequence Propagation Velocity Menu (A6).

The user should consider the following two factors when selecting which SPV estimate to use. First, the propagation velocities calculated for each electrode pair have different resolutions because of the different distances between electrode pairs, and this is not taken

into consideration when the average SPV estimate is used. That is, the estimate for each pair is simply averaged without any weighting based on the error due to discretization. Second, the confidence index presented for each estimate is an indication only of the cross-correlation, i.e., the degree of similarity between the signals detected on the two selected electrodes. This means that the confidence index does not in any way represent the error due to discretization.

4.3.4 Kymograph

A kymograph is a single-image representation of a dynamic process. The Kymograph (A7) in the Main GUI shows the voltage signals for the selected electrodes, with the horizontal axis of the kymograph in this GUI representing the recording time and each row of pixels representing a single electrode. The top row represents the start (most proximal) electrode in in the Electrode Selection Menus (D7), and the bottom row represents the end (most distal) electrode.

The intensity of each pixel corresponds to the value of the voltage signal at each sampling point. The recorded voltages on all of the electrodes shown in the plotting window are rescaled so that all values lie in the range of [0, 1], and these values are translated into pixel intensities in the color map. Thus, when an AP propagates along an axon near a given electrode, the kymograph typically shows the characteristic triphasic extracellular spike as a sequence of pixels that are first bright, then dark, then bright again. In some cases, not all phases are sufficiently large to provide an observable intensity change; for example, a number of spikes in the example data shown in this manual were recognizable only from their negative phase, and thus only a low-intensity region was observable for some electrodes, as with electrode B11 (bottom row of pixels) in Fig. 4.2.

With all selected electrodes plotted, the progression of this triphasic intensity is readily observable from top to bottom, providing an easily interpretable indication of the following features: (i) the existence of a traveling AP, (ii) the direction and speed of propagation of the AP, (iii) the duration of the spike waveforms recorded on each of the electrodes, and (iv) the relative magnitudes of the peak voltages on each electrode. In the example shown in Fig. 4.2, anterograde propagation can be seen as the dark pixels on each electrode traveling from top to bottom (i.e., from B8 to B11) as time progresses.

The user can interact with the Kymograph (A7) to obtain a manual estimate of the propagation velocity. Clicking the “Manual PV calculation” button in the Kymograph Propagation Velocity Tools (A8) allows the user to draw a line on the kymograph. The end points of the line can be adjusted once it has been drawn by clicking and dragging. As shown in the example in Fig. 4.2, the end points of the line should align with the uppermost end of the pixel in the upper electrode selected for manual calculation to the lowermost end of the pixel in the lower electrode. This ensures that the calculated distance between the two points on the kymograph corresponds to the actual spacing between the electrodes. Once the line is in the desired position, double clicking it finalizes the calculation, and the resulting manual estimate is output in the text in the Kymograph Propagation Velocity Tools (A8).

4.3.5 Audio playback

The user may also interact with the recorded signals via an Audio Playback (A9) function. This feature takes advantage of the ability of the human auditory system to recognize sound patterns and is related to the fact that many electrophysiologists rely on the audio outputs of amplifiers to detect spikes. The voltage data are normalized and converted to sound intensities, and each electrode is assigned a tone (single audio frequency). Based on this conversion, when the magnitude of the deviation of the voltage signal from the mean increases on a given electrode, the tone assigned to that electrode becomes louder. In this way, as an AP propagates from one electrode to the next, the recorded spikes can be heard as a progression in tones over time with the tone frequency sequentially increasing for anterograde propagation and decreasing for retrograde propagation.

A timer is provided to show the user the time in the recording corresponding to the audio playback time. To slow down the playback to a speed at which the spike durations and inter-electrode delays are readily detectable by the human ear, the playback is executed at an audio sampling rate of 2 kHz, and the data are resampled at a rate of 500 times the ratio of the audio sampling rate to the recording sampling rate. This results in an approximately 500-fold time dilation; that is, a time period of 1 ms in the recording corresponds to approximately 0.5 s in the audio playback. Left uninterrupted, the audio playback plays 1 s of the recorded data, corresponding to 500 s of playback time. The user may also choose from a list of different chord options to play different sequences of notes.

The start time for the audio playback can be selected by typing the desired time into the Audio Playback textbox (A9). Clicking the “Start” button begins the audio playback and the timer. The audio playback can also be paused, resumed, and stopped by clicking the corresponding buttons in the Audio Playback (A9) section.

5 Spike Sorting GUI

The Spike Sorting GUI of µSpikeHunter can be opened by clicking the Multi-Spike Analysis Button (A10) in the Main GUI. This opens a new window with plots of spike overlays for each electrode and a collection of tools for spike sorting and source cluster analysis. A source cluster is a collection of sorted propagation sequences considered to arise from the same neurite. This section gives an overview of the objects in the Spike Sorting GUI and describes the spike sorting process and results.

***Note**: The Spike Sorting GUI is tied to the Main GUI. Closing the Main GUI while the Spike Sorting GUI is open will also close the Spike Sorting GUI.*

5.1 List of objects in Spike Sorting GUI

This section shows again the labeled diagram of the Spike Sorting GUI with brief descriptions of each labeled object in the diagram. The objects in the Spike Sorting are labeled in orange and numbered S1–S7, and the file saving object is labeled in purple and numbered F4.

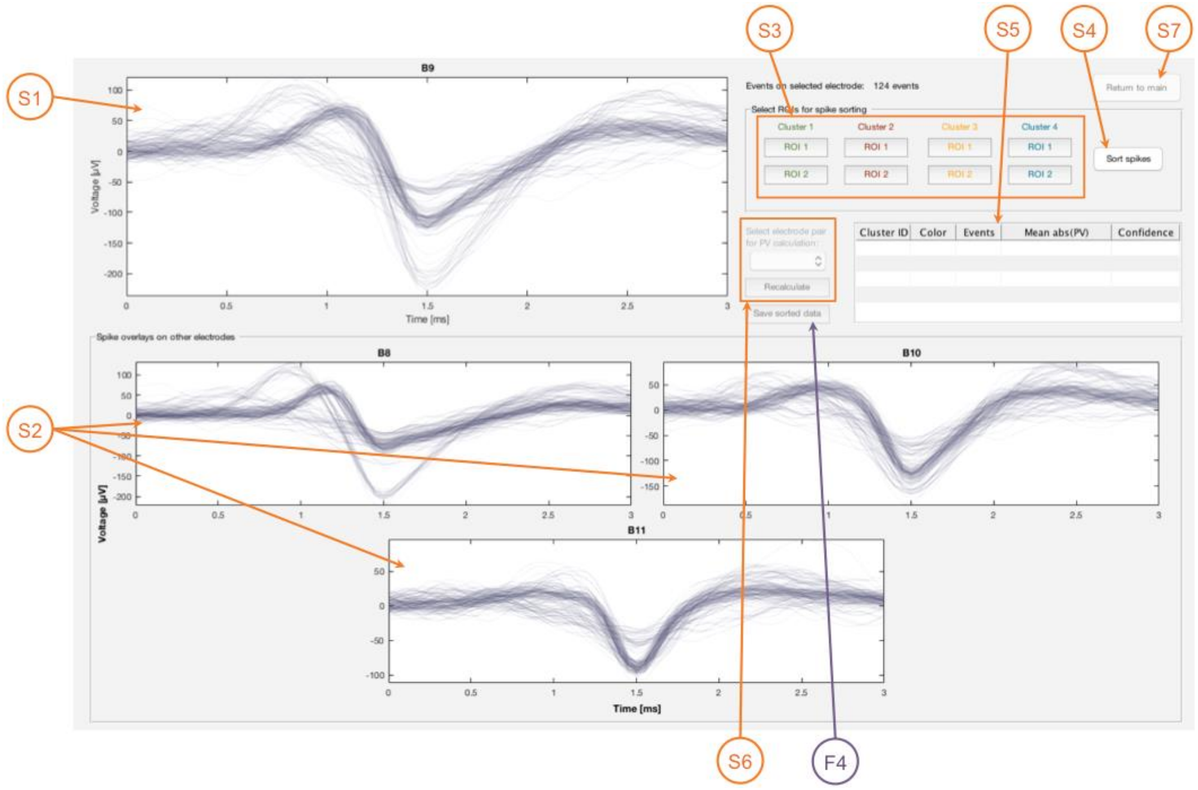

Spike Sorting GUI

(S1) **Event Electrode Spike Overlay Plot**: Shows a plot of all the events on the event electrode detected as part of propagation sequences aligned based on their minima

(S2) **Other Electrode Spike Overlay Plots**: Show plots of events corresponding to the events detected on the event electrode on each of the other electrodes

(S3) **ROI Definition Tools**: Allow the user to draw one or two ROIs for up to four clusters to sort the plotted events

(S4) **Sort Spikes Button**: Sorts the events into source clusters based on the defined ROIs and outputs the results into the Sorting Results Table

(S5) **Sorting Results Table**: Displays the results of the spike sorting, including the cluster ID, plotting color, number of events, and mean propagation speed for each cluster

(S6) **Cluster Propagation Velocity Electrode Selection**: Allows the user to select the electrode pair for calculating the CPV

(S7) **Return to Main GUI Button**: Sends cluster IDs and CPV estimates back to the Event List (A3) in the Main GUI

Saving files

(F4) **Save Sorted Data Button**: Saves a CSV file for each source cluster with the spike times on each electrode, the CPV, the SPV, and the CPV and SPV confidence indices for each propagation sequence in that cluster

5.2 Spike overlays and ROI definition

When it opens, the Spike Sorting GUI shows plots of all of the events detected on the event electrode in the Event Electrode Spike Overlay Plot (S1) and corresponding events on all other electrodes in the Other Electrode Spike Overlay Plots (S2). The number of detected propagation sequences is given beside the Event Electrode Spike Overlay Plot (S1). The events for each electrode are aligned about their peak voltage values (maxima for positive phase detection and minima for negative phase detection).

The user may draw one or two regions of interest (ROIs) on the Event Electrode Spike Overlay Plot (A1) for up to four source clusters for the spike sorting process. An ROI is drawn by clicking one of the ROI buttons for one of the clusters in the ROI Definition Tools (R3). Any combination of ROIs can be drawn in any order; that is, ROI 2 can be drawn for a cluster without first drawing ROI 1, and ROIs for each cluster can be drawn without first defining ROIs for clusters with lower cluster IDs.

Once an ROI has been drawn, the locations of its edges can be adjusted by clicking and dragging. ROIs may also be deleted by right clicking and selecting “Delete” from the menu that appears. If the button for an ROI that has already been drawn is clicked, that ROI is deleted and the user may redraw it.

Spikes are assigned to source clusters sequentially from Cluster 1 (green) to Cluster 4 (blue) and taken out of the sorting pool once they have been assigned a cluster. This means that ROIs should be drawn more selectively for clusters with lower cluster IDs and ROIs for clusters with higher cluster IDs may be drawn to encompass spikes from clusters with lower cluster IDs. An example of this is described in more detail in the next section.

Note: *When drawing an ROI in a region where the signals are rapidly changing (i.e., have a steep positive or negative slope), the user should bear in mind that µSpikeHunter is dealing with discrete data and ensure the height of the ROI is sufficiently large to capture at least one data point for each spike. See Fig. 5.1 on the next page for an example.*

In regions where the voltage is rapidly changing, the recorded voltage at one sampling point is much higher or lower than that at the subsequent sampling point. This means that if the height of the ROI is smaller than the magnitude of this change, one data point may be above the ROI and the next may be below it; thus, even though the plotted trace appears to pass through the ROI, the event would not be assigned to the cluster for that ROI; this is the case for the results in Fig. 5.1(a). Although drawing an ROI with a greater height fixes this problem (Fig. 5.1(b)), the problem is best avoided by drawing ROIs in regions of low change (i.e., near the peaks of the spikes), as in Fig. 5.1(c).

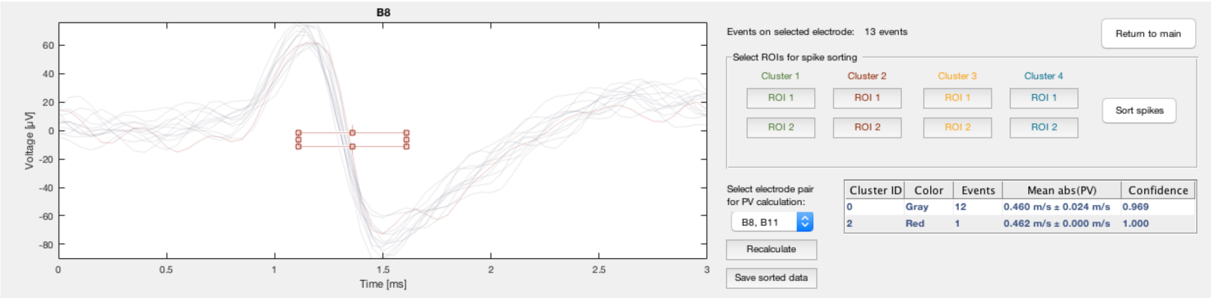

(a) Sorting results for ROI with small height in high-gradient region

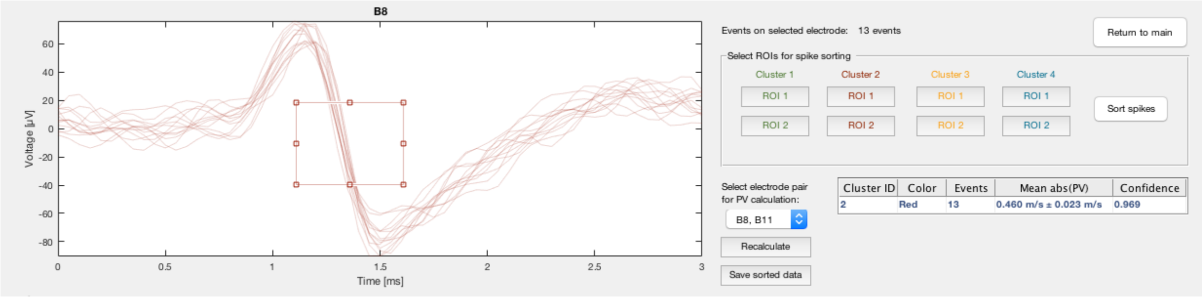

(b) Sorting results for ROI with large height in high-gradient region

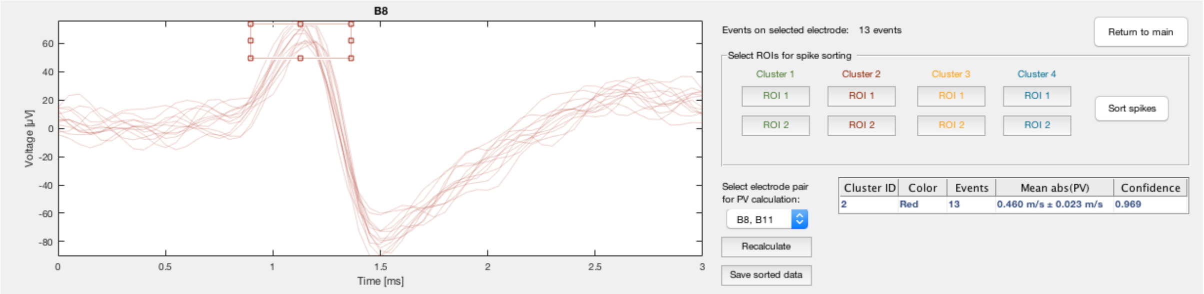

(c) Sorting results for ROI in low-gradient region (recommended)

Fig. 5.1. *Examples of spike sorting results on the event electrode for (a) an ROI with a small height in a region of rapidly changing voltage, (b) an ROI with a large height in a region of rapidly changing voltage, and (b) an ROI drawn near a voltage peak (recommended).*

5.3 Spike sorting process and results

Clicking the Sort Spikes Button (S4) sorts the spikes into source clusters based on the ROIs drawn on the Event Electrode Spike Overlay Plot (S1) and outputs the results into the Sorting Results Table (S5). An example of the spike sorting result is shown in Fig. 5.2.

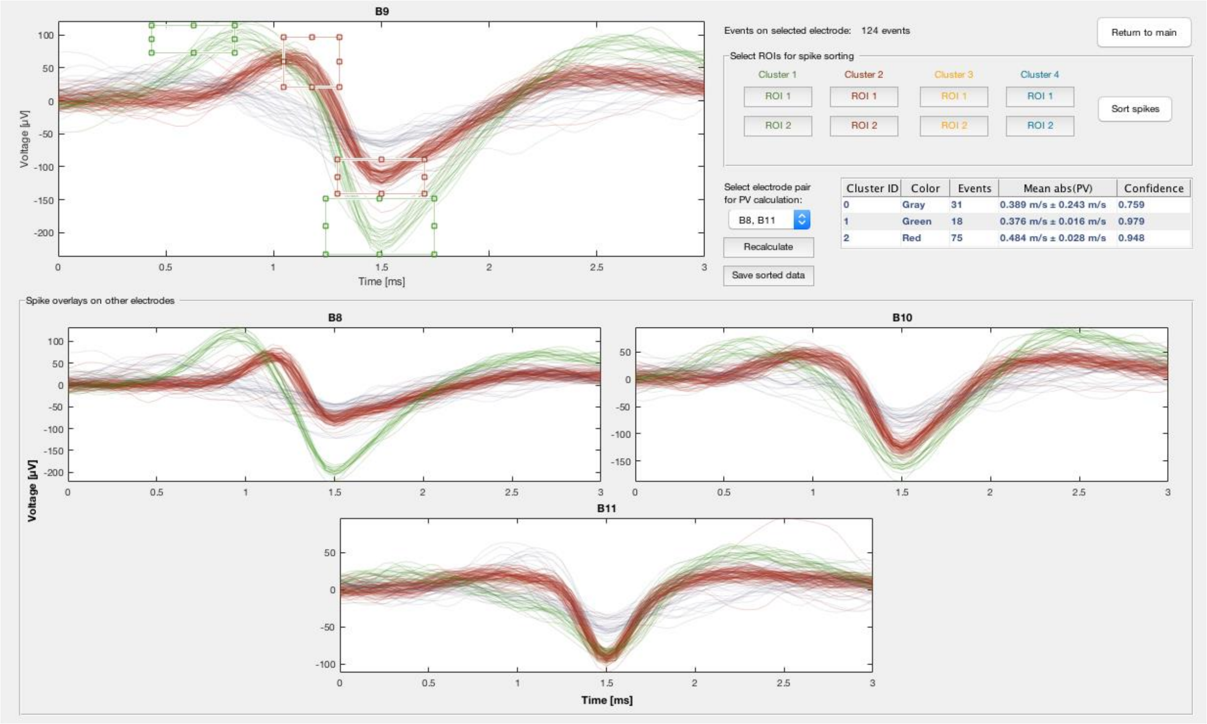

Fig. 5.2. Analysis panel of Main GUI. See the diagram on page 18 for names and labels of analysis objects.

Spikes are assigned to a cluster if the voltage trace passes through all of the ROIs drawn for that cluster and if they have not been sorted to a cluster with a lower cluster ID. The spike sorting algorithm progresses from Cluster 1 (green) to Cluster 4 (blue), and once a spike has been assigned to a cluster, it is removed from the sorting pool. The spikes in the Event Electrode Spike Overlay Plot (S1) and the Other Electrode Spike Overlay Plots (S2) are colored to correspond to the cluster to which they have been assigned.

Any spikes not assigned to Clusters 1–4 after the sorting process are assigned to Cluster 0 and remain the same color as in the original plot. Cluster 0 may be considered to correspond to a separate neurite or as a cluster of unsorted events. The ROIs remain on the event electrode plot after the spikes have been sorted, and the user may adjust or delete the ROIs and re-sort the spikes as desired.

In the example shown in Fig. 5.2, two source clusters, Clusters 1 (green) and 2 (red), have been defined using two ROIs each. Note that although a number of spikes in Cluster 1 pass through both ROIs for Cluster 2, these spikes are still assigned to Cluster 1. This is because of the sequential nature of the sorting process described here and in Section 5.2: once the green spikes are assigned to Cluster 1, they are removed from the sorting pool and are no longer available to be assigned to Cluster 2.

The Sorting Results Table (S5) is then populated with the spike sorting results. The first two columns give the cluster ID and the corresponding color for that cluster. The third column gives the number of events in each cluster. The fourth and final columns give the average cluster propagation velocity (CPV) estimate for the cluster and the corresponding average confidence index. The CPV is calculated based on the cluster-based redefinitions of the event times, which are obtained using the procedure described in the next section, and the confidence index is an indication of the intra-cluster intra-electrode similarity of the spike shapes on the two electrodes selected for CPV estimation.

5.4 Cluster propagation velocity estimation

This section describes the method used to calculate the CPV estimate. First, each spike is realigned by maximizing the cross-correlation with every other event in the cluster. The new event time for each event in a cluster is then defined as the time at which the sum of all of the realigned traces is minimized or maximized (for negative or positive phase event detection, respectively). For a more detailed explanation of this process, the user is referred to the paper on this program []. Whereas using the timing of the peak voltage for each event would yield an unreliable approximation of the event time, minimizing or maximizing the sum of the aligned events yields a more accurate estimation.

This event time estimation procedure is performed for each electrode, and the propagation velocity of each propagation sequence is then defined based on the spike times on the two most distant electrodes. However, the user may choose to calculate the propagation velocity using any pair of electrodes by selecting the desired pair from the Cluster Propagation Velocity Electrode Selection (S6) and clicking the “Recalculate” button. The mean propagation speed for each cluster is then calculated and presented in the Sorting Results Table (S5) along with the standard deviation. If the standard deviation of the CPV is equal to or greater than the average CPV, the result is given as “Undefined” to indicate that the propagation sequences in the cluster likely do not correspond to a single neurite.

The Sorting Results Table (S5) also gives a confidence index for the CPV estimate. This confidence index is based on the spike realignment process. As stated above, the cross-correlation between each event and every other event on the same electrode sorted into the same cluster is calculated to perform the realignment. The maximum value of the autocorrelation-normalized cross-correlation is obtained for each of these cross-correlations and averaged for each event. Thus, each event on each electrode is assigned an event-based confidence index in the range [0, 1] that indicates the confidence of its realignment time based on the intra-electrode intra-cluster cross-correlations. When an electrode pair is selected for the CPV estimation, the CPV confidence index for each propagation sequence is obtained as the average of the two event-based indices for the events in the propagation sequence on the selected electrodes. This confidence index indicates the degree of similarity of the spike shapes on the two electrodes selected for CPV estimation for spikes within the same sorting cluster.

It should be noted that the CPV and the SPV are estimating different quantities: the CPV essentially estimates the delay between the peak voltages arriving on the two selected electrodes based on the timing of the peak voltage of a “master spike” for each cluster on each electrode, whereas the SPV estimates the lag for a single propagation sequence based on matching the waveforms recorded on the selected electrodes. Thus, if the waveforms on the two selected electrodes are different, the resulting propagation velocity estimates may also differ because the lag that yields the highest cross-correlation for the SPV may not correspond to the delay between the peak voltages on the two electrodes. It should also be noted that the CPV is more robust against noise than the SPV.

5.5 Returning results to Main GUI

Clicking the Return to Main GUI Button (S7) or closing the Spike Sorting GUI after spike sorting has been conducted updates the Event List (A3) in the Main GUI with the cluster ID and the CPV estimate for each event. An example of the appearance of the analysis panel with this updated list is shown in Fig. 5.3.

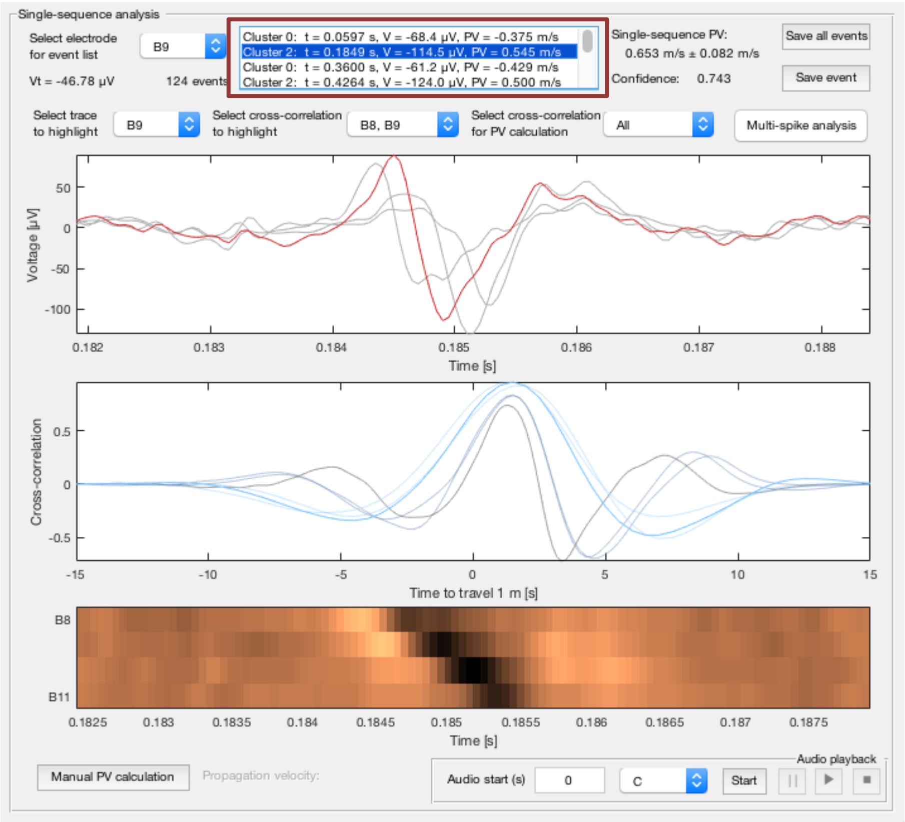

Fig. 5.3. Analysis panel of Main GUI with the Event List (A3) updated to include the cluster ID and CPV estimate for each event.

6 Saving files

The analysis results obtained using µSpikeHunter can be exported in different formats for further external analysis using a number of export buttons in the Main and Spike Sorting GUIs. This section presents descriptions of the four types of files that can be exported from µSpikeHunter.

6.1 Information contained in filenames and data file headers

All files are saved with filenames and headers containing information about the analysis so that the steps to analyze the data can be repeated to obtain the same results. The base filename and header for each type of saved file contains the following information: the filename of the recorded data file being analyzed, as given in the Filename Textbox (D2); the start and end electrodes selected for analysis, as defined in the Electrode Selection Menus (D7); the start time and duration selected for analysis, as defined in the Analysis Start Time and Duration Menus (D6); and the number of standard deviations selected for the event detection threshold, as defined in the Event Detector Parameters (D10).

Additional information is added for each specific type of saved file, as will be described in the corresponding sections here. An example of how the information is organized in the base filename is presented below. The same information is also included in the file header. In the case of a custom setup, the electrodes are simply assigned numbers for the second item in the list (e.g., "1-4" in the case of four electrodes), the start time and duration are not included in the filename, and the file header does not contain any information about the electrodes or analysis times.

Example: filename1_B1-B4_0s_60s_-5sig_[...].dat

- *Name of recording file*: filename1
- *Electrodes selected for analysis*: electrodes B1 to B4 (i.e., B1, B2, B3, B4)
- *Start time of analysis*: 0 s
- *Analysis duration*: 60 s
- *Event detection threshold*: 5 standard deviations, negative phase detection

6.2 All analyzed data

Clicking the Save Analysis Data Button (F1) in the Main GUI saves a CSV file with the time stamps in milliseconds and voltage data in microvolts for each selected electrode in the time range selected for analysis. This allows the user to analyze all the data selected for analysis using software other than µSpikeHunter or Multi Channel Analyzer. The organization of the data in the CSV file is the same as that required for custom setup recording files (see Fig. 3.1). The base filename described in Section 6.1 is appended with the word "data" for this type of file.

6.3 All detected propagation sequences

Clicking the Save All Events Button (F2) in the Main GUI saves a CSV file listing all detected propagation sequences in the analyzed data. Each pair of columns giving the time in milliseconds at which the peak voltage value occurred and the peak voltage value in microvolts for the set of events on each electrode in each propagation sequence. The data are organized in the CSV file as shown in Fig. 4.2. The base filename described in Section 6.1 is appended with the word "events" for this type of file.

	B1		B2		B3		B4	
	Time (ms)	Peak (µV)	Time (ms)	Peak (µV)	Time (ms)	Peak (µV)	Time (ms)	Peak (µV)
	1	2	3	4	5	6	7	8
1	184.7000	-69.1482	184.9000	-114.4701	185.1000	-130.9210	185.3000	-95.3324
2	426.1500	-74.9608	426.3500	-124.0116	426.6000	-120.3924	426.7500	-81.2121
3	547.4000	-59.0584	547.6000	-88.0666	547.8500	-104.7093	548	-83.1314
4	587.4000	-221.7842	587.6000	-226.4727	587.8500	-165.1386	588.1500	-92.6454
5	904.7500	-62.2663	905.0500	-109.7543	905.2000	-136.4320	905.4000	-103.1739
6	1.3728e+03	-84.6942	1.3730e+03	-123.0794	1.3733e+03	-143.2042	1.3734e+03	-94.8937
7	3.7134e+03	-83.6249	3.7136e+03	-118.5828	3.7139e+03	-130.7839	3714	-102.3788
8	4.4322e+03	-72.1916	4.4324e+03	-110.6316	4.4325e+03	-136.4868	4.4327e+03	-94.7840
9	6.0606e+03	-74.4673	6.0608e+03	-113.3734	6061	-106.8479	6.0612e+03	-92.7003
10	6.6605e+03	-94.2631	6.6607e+03	-129.0565	6.6609e+03	-143.8897	6.6611e+03	-95.4421
11	6.9086e+03	-78.4429	6.9088e+03	-122.3665	6909	-128.9743	6.9092e+03	-87.8473
12	7.6668e+03	-86.2570	7.6671e+03	-131.4693	7.6673e+03	-128.3985	7.6675e+03	-97.7452
13	7.9975e+03	-192.3921	7.9977e+03	-221.3455	7998	-145.5622	7.9983e+03	-88.2860
14	8.1491e+03	-93.3857	8.1493e+03	-108.3285	8.1496e+03	-116.0330	8.1497e+03	-97.4162
15	8.5761e+03	-70.6013	8.5763e+03	-116.2249	8.5766e+03	-124.6696	8.5767e+03	-85.3797

Fig. 6.1. Organization of file listing the times (ms) and amplitudes (µV) of the events in all propagation sequences. The columns are labeled to correspond to the electrodes in one of the microchannels in the example µEF configuration shown in Fig. 1.1.

6.4 Traces for a single propagation sequence

Voltage traces for a single propagation sequence can be exported by clicking the Save Event Button (F3) in the Main GUI. The created file contains the voltage trace data for the selected event in the Event List (A3) with the same time window as shown in the Voltage Trace Plot (A4). The file is a CSV file with the first column giving the time stamps and the second to last columns giving the recorded voltage at each sampling time on the selected electrode. This file can be used to make plots of propagation sequences with the desired format using external software. The data are organized in the CSV file as shown in Fig. 6.2.

	Time (ms)	B1 (µV)	B2 (µV)	B3 (µV)	B4 (µV)
	1	2	3	4	5
1	181.8500	-2.1660	3.5918	7.6770	11.7623
2	181.9000	4.2772	8.4173	9.1302	9.2399
3	181.9500	8.0609	11.4333	7.5674	4.4417
4	182	8.9657	13.4348	5.1820	2.8241
5	182.0500	9.2124	14.5590	5.7029	6.5529
6	182.1000	8.5818	13.1058	8.5544	12.0913
7	182.1500	6.0868	9.1576	10.2269	13.8187
8	182.2000	2.9337	4.8804	10.1447	11.5704
9	182.2500	1.9193	3.2902	10.9672	9.2124
10	182.3000	2.0289	3.7288	12.8316	8.6641

Fig. 6.2. First 10 samples of voltage traces for a single propagation sequence saved to a CSV file. The file lists the time stamps (ms) and the voltage (µV) recorded on each electrode at that time over the same window as shown in the Voltage Trace Plot (A4) for the selected event in the Event List (A3). The columns are labeled to correspond to the electrodes in one of the microchannels in the example µEF configuration shown in Fig. 1.1.

The additional information added to the filename and header for this type of file is the number of the saved event. For example, filename1_B1-B4_0s_60s_-5sig_**seq7**.dat corresponds to the single-sequence file for the seventh propagation sequence detected in the analyzed time range, and the event number is also given in the header. All other information included in the filename and header is as described in Section 6.1.

6.5 Spike sorting results

The spike sorting results can be saved to CSV files by clicking the Save Sorted Data Button (F4) in the Spike Sorting GUI. This button saves a CSV file for each source cluster, including Cluster 0. For example, for the spike sorting results shown in Fig. 5.2, clicking the Save Sorted Data Button (F4) would create three CSV files for Clusters 0, 1, and 2. The information included in each file is as follows for each propagation sequence in the cluster: the index of the propagation sequence, the time at which the spike arrives on the event electrode, the time at which the spike arrives on each of the electrodes, the CPV estimate and its confidence index, and the SPV and its confidence index. The CPV and SPV estimates are obtained using the electrode pairs selected in the Cluster Propagation Velocity Electrode Selection Menu (S6) and the Single-sequence Propagation Velocity Menu (A6), respectively. See Sections 5.4 and 4.3.3 for explanations of the CPV and SPV calculations. The data are organized in the CSV file as shown in Fig. 6.3.

	No.	B2 time (ms)	B1 time (ms)	B2 time (ms)	B3 time (ms)	B4 time (ms)	CPV	Conf.	SPV	Conf.
	1	2	3	4	5	6	7	8	9	10
1	8	587.6000	587.4000	587.6000	587.8500	588.1500	0.4000	0.9628	0.5000	0.7361
2	18	7.9977e+03	7.9975e+03	7.9977e+03	7.9979e+03	7.9982e+03	0.4286	0.9666	0.4615	0.7844
3	28	1.4090e+04	1.4090e+04	1.4090e+04	1.4090e+04	1.4090e+04	0.4000	0.9757	0.4615	0.8214
4	32	14601	1.4601e+04	14601	1.4601e+04	1.4602e+04	0.4000	0.9561	0.4615	0.6877
5	34	1.4639e+04	1.4639e+04	1.4639e+04	1.4640e+04	1.4640e+04	0.4286	0.9704	0.5455	0.7367
6	35	14812	1.4812e+04	14812	1.4812e+04	1.4813e+04	0.4000	0.9698	0.5000	0.8382
7	37	1.4929e+04	1.4928e+04	1.4929e+04	1.4929e+04	1.4929e+04	0.4000	0.9657	0.4286	0.8823
8	41	1.5270e+04	1.5269e+04	1.5270e+04	1.5270e+04	1.5270e+04	0.4286	0.9726	0.5455	0.7902
9	47	1.5621e+04	1.5621e+04	1.5621e+04	1.5622e+04	1.5622e+04	0.4000	0.9560	0.4286	0.8332
10	51	1.6038e+04	1.6038e+04	1.6038e+04	1.6038e+04	1.6039e+04	0.4000	0.9747	0.5000	0.7824

Fig. 6.3. Organization of the sorting results file. The first column gives the event number, the second gives the time of the peak voltage on the event electrode, the next columns give the times of the peak voltages on all electrodes, and the final four columns give the CPV and SPV estimates with their corresponding confidence indices. The columns are labeled to correspond to the electrodes in one of the microchannels in the example µEF configuration shown in Fig. 1.1 with B2 as the event electrode.

The additional information added to the filename and header for this type of file is the event electrode, the electrode pairs used for the CPV and SPV estimates, and the cluster ID. For example, filename1_B1-B4_0s_60s_-5sig_**selB2_CPVB1-B4_SPVall_clst1**.dat corresponds to the sorting results file for all propagation sequences assigned to Cluster 1 with sorting performed on electrode B2, and the CPV and SPV estimates in the file were calculated using electrodes B1 and B4 and the average of all electrode pairs, respectively. The same information is included in the file header. All other information included in the filename and header is as described in Section 6.1.

7 Glossary of terms

A number of terms have been adopted for ease of explanation in this user manual. This section provides the definitions of these terms for reference by the user.

Cluster (or source cluster): When spike sorting is performed, the propagation sequences obtained based on event detection applied to a single electrode are sorted into source clusters. Clusters of propagation sequences are considered to arise from different APs propagating along the same axon.

Cluster identification (cluster ID): The cluster ID is a number ranging from 0 to 4 that corresponds to the cluster into which a given spike has been sorted.

Cluster propagation velocity (CPV): The CPV is an estimate of the propagation velocity obtained based on the alignment of all spikes sorted into a cluster, as described in Section 5.3.

Event: An event is any portion of a voltage trace recorded on an electrode in which the voltage signal surpasses the user-defined threshold for event detection. Events may be either electrode noise, instrument noise, or spikes.

Event electrode: The term “event electrode” is used to refer to the electrode selected to populate the list of events in the Main GUI. In the Spike Sorting GUI, the ROIs used for spike sorting are drawn on the spike overlay plot for the event electrode.

Graphical user interface (GUI): A GUI is a computer program that allows the user to interact with a computer through graphical elements, such as buttons, text boxes, and plots. µSpikeHunter consists of two GUIS: the Main GUI and the Spike Sorting GUI.

Instrument noise: The term “instrument noise” in this manual is used to refer to (usually large-amplitude) fluctuations in the voltage signal that are produced by disturbances in the environment of the recording system. These disturbances generally have approximately the same timing and shape across all recording electrodes.

Microelectrode array (MEA): An MEA consists of multiple microelectrodes embedded in a substrate. MEAs can be used to record spikes as well as evoke neuronal activity.

Microelectrode–microfluidic (µEF) device: A µEF device is a compartmentalized cell culture device that is composed of a microfluidic device with integrated microelectrodes for electrophysiology. In its simplest configuration, it is composed of a microfluidic device aligned with and mounted on a MEA. The dimensions of the microchannels in µEF devices are designed such that somata are excluded from the microchannels whereas axons are able to grow through the channels. See Fig. 1.1.

Propagation sequence (or sequence): A propagation sequence is defined as a collection of temporally linked events recorded on all electrodes selected for analysis. Sequences are considered to be representative of propagating APs.

Region of interest (ROI): An ROI is a rectangular region in event time–voltage space defined by the user in the Spike Sorting GUI. One or two ROIs can be defined for up to four clusters to perform spike sorting.

Signal noise: The term “signal noise” in this manual is used to refer to the combination of electrode noise and instrument noise that accompanies any electrical recording.

Single-sequence propagation velocity (SPV): The SPV is an estimate of the propagation velocity obtained based on the cross-correlation of the voltage traces in a single propagation sequence as described in Section 4.3.3.
